# Subarctic singers: Humpback whale (*Megaptera novaeangliae*) song structure and progression from an Icelandic feeding ground during winter

**DOI:** 10.1371/journal.pone.0210057

**Published:** 2019-01-23

**Authors:** Edda E. Magnúsdóttir, Rangyn Lim

**Affiliations:** 1 The University of Iceland’s Research Center in Húsavík, Húsavík, Iceland; 2 Department of Life and Environmental Sciences, University of Iceland, Reykjavík, Iceland; McGill University, CANADA

## Abstract

Humpback whale songs associated with breeding behaviors are increasingly reported outside of traditional low latitude breeding grounds. Songs from a subarctic feeding ground during the winter were quantitatively characterized to investigate the structure and temporal changes of the songs at such an atypical location. Recordings were collected from 26. January to 12. March, 2011, using bottom mounted recorders. Humpback songs were detected on 91% of the recording days with peak singing activities during 9.–26. February. The majority of the recordings included multiple chorusing singers. The songs were characterized by a) common static themes which transitioned consistently to predictable themes, b) shifting themes which occurred less predictably and c) rare themes. A set median sequence was found for four different periods (sets) of recordings (approximately 1 week each). The set medians were highly similar and formed a single cluster indicating that the sequences of themes sung in this area belonged to a single cluster of songs despite of the variation caused by the shifting themes. These subarctic winter songs could, thus, represent a characteristic song type for this area which is comparable to extensively studied songs from traditional low latitude breeding grounds. An increase in the number of themes per sequence was observed throughout the recording period including minor changes in the application of themes in the songs; indicating a gradual song progression. The results confirm that continual singing of sophisticated songs occur during the breeding season in the subarctic. In addition to being a well-established summer feeding ground the study area appears to be an important overwintering site for humpback whales delaying or canceling their migration where males engage in active sexual displays, i.e. singing. Importantly, such singing activity on a shared feeding ground likely aids the cultural transmission of songs in the North Atlantic.

## Introduction

Humpback whales (*Megaptera noveangliae*) are a well-studied migratory species, travelling annually between summer feeding areas in subpolar waters where they exploit the rich nutritional resources, and to tropical wintering areas to breed [1]. Females give birth and mate during the winter where the gestation period is approximately 12 months [2]. Females commonly give birth every two years, however, that can vary and likely depends on body condition and other ecological factors [3, 4]. A high degree of maternally-directed site fidelity is observed when they migrate between these summer and winter grounds [5, 6]. In the North Atlantic, the feeding grounds stretch from the Arctic waters of N-Norway and Jan Mayen to the east and Greenland, St. Lawrence and towards the mid-latitudes of the Gulf of Main to the west [6]. Coastal Icelandic waters, located in the central North Atlantic, are also common subarctic summer feeding grounds for humpback whales as well as other mysticete cetaceans, including blue whales (*Balaenoptera musculus*), fin whales (*Balaenoptera physalus*) and minke whales (*Balaenoptera acutorostrata*) [7]. The feeding period of humpback whales in the North Atlantic generally extends from April (spring) and until November (early winter) with the highest rate of sightings during the peak summer months, i.e. June to August [8]. Southbound migration usually starts in early winter during October/November and extends into the winter where whales generally arrive on their breeding grounds during mid- to late February [9] and even in spring (March-May) [10]. Several studies have provided evidences of possible overwintering of humpback whales and other mysticete species on their feeding grounds. Humpback whales and fin whales were sometimes found overwintering in high latitude feeding grounds of the Arctic as well as in the Antarctic [11, 12]. In the Southern Hemisphere, Van Opzeeland *et al*. [12] observed an acoustic presence of humpback whales throughout austral winter and summer, indicating that they are overwintering in the area despite the presence of accumulating sea ice. Therefore, migration appears to vary by individuals and is evidently affected by multiple ecological factors.

Studies have shown that the location of feeding grounds, sex, age, and reproductive status of an individual whale can affect the timing of migration [1, 9, 13]. Humpback whales originating from the eastern and central North Atlantic feeding grounds, such as Iceland and Norway, have been observed to arrive at their westerly Caribbean breeding grounds (off the Dominican Republic and Puerto Rico) later (end of February) than whales migrating from western feeding grounds (early to mid-February), such as from the east coast of North America [9]. To date, only whales from the eastern and central feeding grounds have been sighted in the Cape Verde area as well as in the Guadeloupe area, a particular easterly breeding assembly in the Caribbean’s [10, 14]. Humpback whales rarely arrive in these two areas before March, with the mean sighting dates in the Cape Verde area and Guadeloupe estimated to be in early to mid-April [10]. Despite a mere 1000 km distance between the Guadeloupe and the Dominican Republic breeding grounds, there appears to be very little interaction between individuals visiting the two breeding areas [10].

### Singing outside of traditional breeding grounds

**Fig 1:**
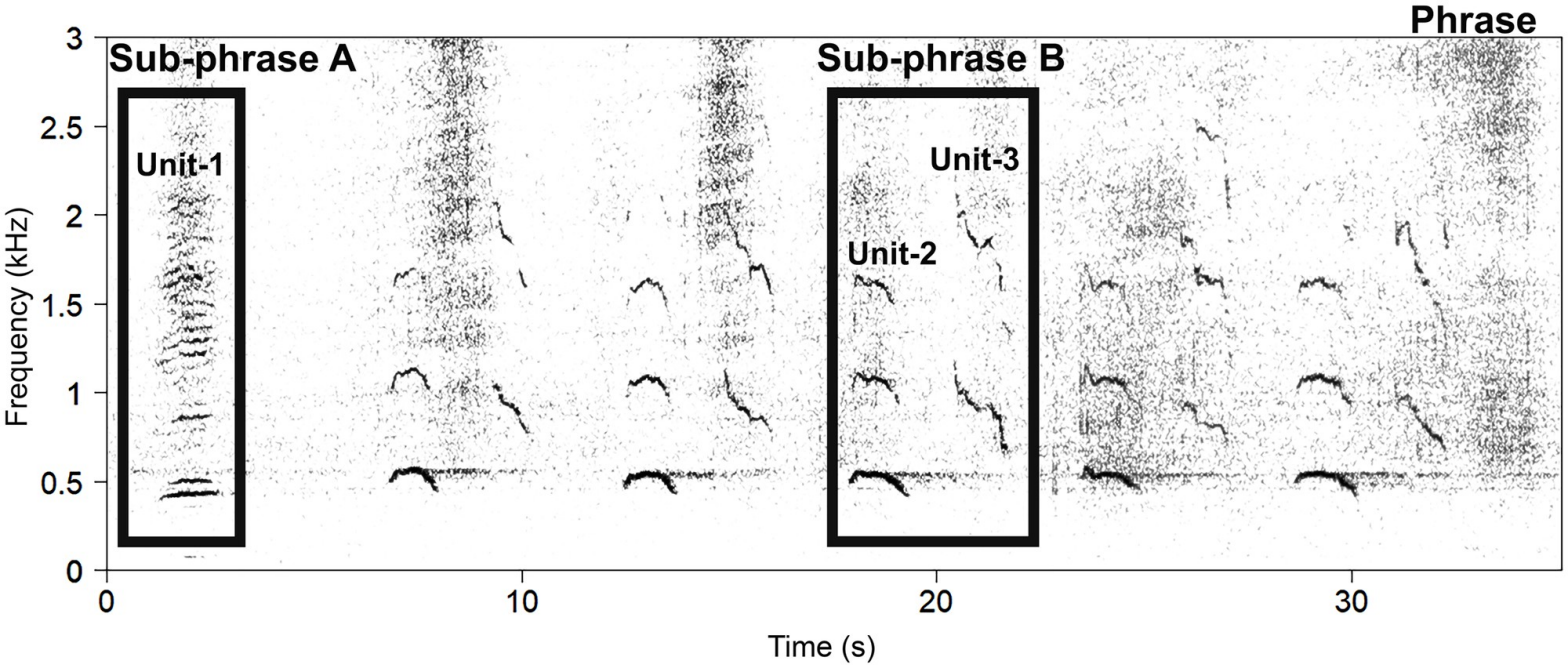
Spectrogram representation of a typical humpback whale song phrase observed during this study. This phrase is composed of two sub-phrase types (referred to here as A and B). Sub-phrase A is sung at the beginning of the phrase and is composed of a single unit. Sub-phrase B is composed of two different unit types and is repeated throughout the phrase.

A particular behavior strongly associated with breeding in humpback whales on low latitude breeding grounds is singing. To date, this behavior has only been observed from males and linked with mating and male-male social organization [15–17]. In the northern hemisphere, occurrence of songs on low latitude breeding grounds have been shown to increase between mid-February and mid-March [18]. This coincides with the female ovulation period, increased testis weight [19–21] and agonistic behavior of male humpback whales, thus, suggesting that these songs have a role in reproduction [16, 22–24]. Social and feeding calls vocalized by both males and females do not have the distinct repetitive structures or patterns and consequently not characterized as songs [16, 25, 26]. Humpback whale songs are characterized by high intensity vocal signals ranging from low to mid-frequencies between at least 8 Hz and 10 kHz [27, 28], and at source levels between at least 151 and 173 dB re 1μPa at 1m [29]. Although descriptions of humpback whale songs vary across literature and delineation methods, the essential foundation for song characterization is generally based on the criteria first proposed by Payne and McVay [30]. The hierarchical song is characterized by the shortest, most basic element in the song called a ‘unit’ which combine to form ‘sub-phrases’ and ‘phrases’ (Fig 1). These phrases are repeated in succession to form ‘themes’ that, when sung continuously, form a ‘song session’.

Humpback whale songs are a well-studied behavioral phenomena; however, a growing body of literature challenges what is traditionally understood as a typical singing and migratory behavior. Magnúsdóttir *et al*. [31] recently reported that individuals acoustically detected in Iceland’s feeding grounds engage in singing during the winter until at least mid-March. The recent findings on late migration patterns, suggest that the North Atlantic humpback whales recorded singing in Iceland are not leaving their high latitude feeding grounds until considerably later than previously reported [8, 9]. Therefore, the humpback whales that remain in Iceland’s feeding ground until mid-March could still arrive on time to their breeding grounds in late spring given that their average travel speed is around 4–4.5 km/h [32, 33] and the distance to the Cape Verde and the Caribbean breeding grounds is around 6000 km. The extended stay in feeding grounds and active singing could allow them to build up energy reserves and increase the possibility of successful mating. Another plausible explanation could be that some of the whales are overwintering and the singing behavior recorded in the subarctic represents non-migrating whales that remain in the prey abundant coastal waters of Iceland throughout the year.

Although the recordings of humpback whale singing activity indicates male presence, it is possible that female humpback whales are also overwintering to avoid the energetically costly migrations [34–36]. In addition to the recent recordings of singing during winter in the subarctic feeding grounds of Iceland [31, 37], humpback whales have been discovered singing along migration routes and at mid-latitude feeding grounds in the North Atlantic during shoulder seasons (i.e., spring and autumn) [38–41] and to some extent during winter [40]. Songs have also been recorded during the austral fall in the Antarctic [42]. In contrast to these findings, however, the most active singing behavior recorded in the feeding grounds of Iceland did not occur during a typical shoulder season but later in the winter [31, 37] during what has been estimated to be the peak of the humpback whale’s breeding season [12–15].

These findings raise questions as to what advantages could be gained from singing away from traditionally known breeding grounds. The behavioral flexibility suggests that singing in these areas could offer a positive trade-off strategy for late migrating individuals and individuals that overwinter in an area with available prey and possible mating opportunity [31, 37]. Another possibility is that the breeding season of this species could stretch further into spring than previously reported and that the singing period in Iceland is simply a shoulder season. Garland *et al*. [43] hypothesized that different breeding populations meeting and singing “off-season” on feeding grounds could also allow for the rapid horizontal cultural transmission of songs.

Cultural transmission is the social learning and sharing of information or behaviors between conspecifics within a population or subpopulation [44, 45]. Cultural traits can change the way in which individuals interact with their environment within and over generations, directly and indirectly affecting feeding success, survival rates, and fitness [46]. Different modes of cultural transmission exist within the humpback whale species and can include both vertical (parent-offspring) and horizontal transmission. Humpback whale songs are constantly changing within a population over time, and these gradual changes are recognized as cultural evolution but revolution when there is a rapid replacement of a complex song over a period of less than two years [47]. The songs are learned through horizontal cultural transmission across unrelated individuals where songs usually evolve gradually. A population will, therefore, usually conform to singing similar dialects or song types within a shared ocean basin [48–50]. Differences begin to appear and increase with distance between populations [51, 52] but are distinctly different between geographically isolated populations [53]. Three possible mechanisms for cultural transmission were hypothesized by Payne and Guinee [50] where song sharing could occur, i.e. 1) the movement of individuals from one breeding population to another between seasons, 2) within season movement of individuals between breeding population and 3) song exchange on a shared feeding ground or migration route. Song similarity has been described between breeding grounds in the North Atlantic, i.e. the Caribbean’s and the Cape Verde Islands [53], suggesting that song exchange could occur in Icelandic waters before the whales reach their two separate breeding grounds.

By further expanding the findings in Magnúsdóttir *et al*. [31], this study seeks to find evidence of whether the songs examined during the breeding season on an Icelandic subarctic feeding ground could serve as mating displays and consequently used as a mode of cultural transmission for humpback whale songs in the North Atlantic. That is done with a comprehensive description of the Icelandic songs and their progression during a single winter season including investigation of the occurrence of chorusing events which may indicate a lekking aggregation of males. If singing in this subarctic region resembles the singing behavior on a traditional low-latitude breeding ground, it is likely that subarctic songs have a role in the humpback whale mating system by 1) starting the song exchange between individuals while on a feeding ground and thus aiding the cultural transmission of songs to different breeding grounds and 2) possibly providing mating opportunities outside of traditional breeding grounds.

Importantly, these findings will provide new information about the singing behavior of humpback whales in high-latitude regions. Thus, further expanding our knowledge on the behavioral plasticity and life-history strategy of this large baleen whale species.

## Methods

The University of Iceland’s Research Centre in Húsavík and The Marine and Freshwater Research Institute permitted the research.

### Acoustic recordings

**Fig 2:**
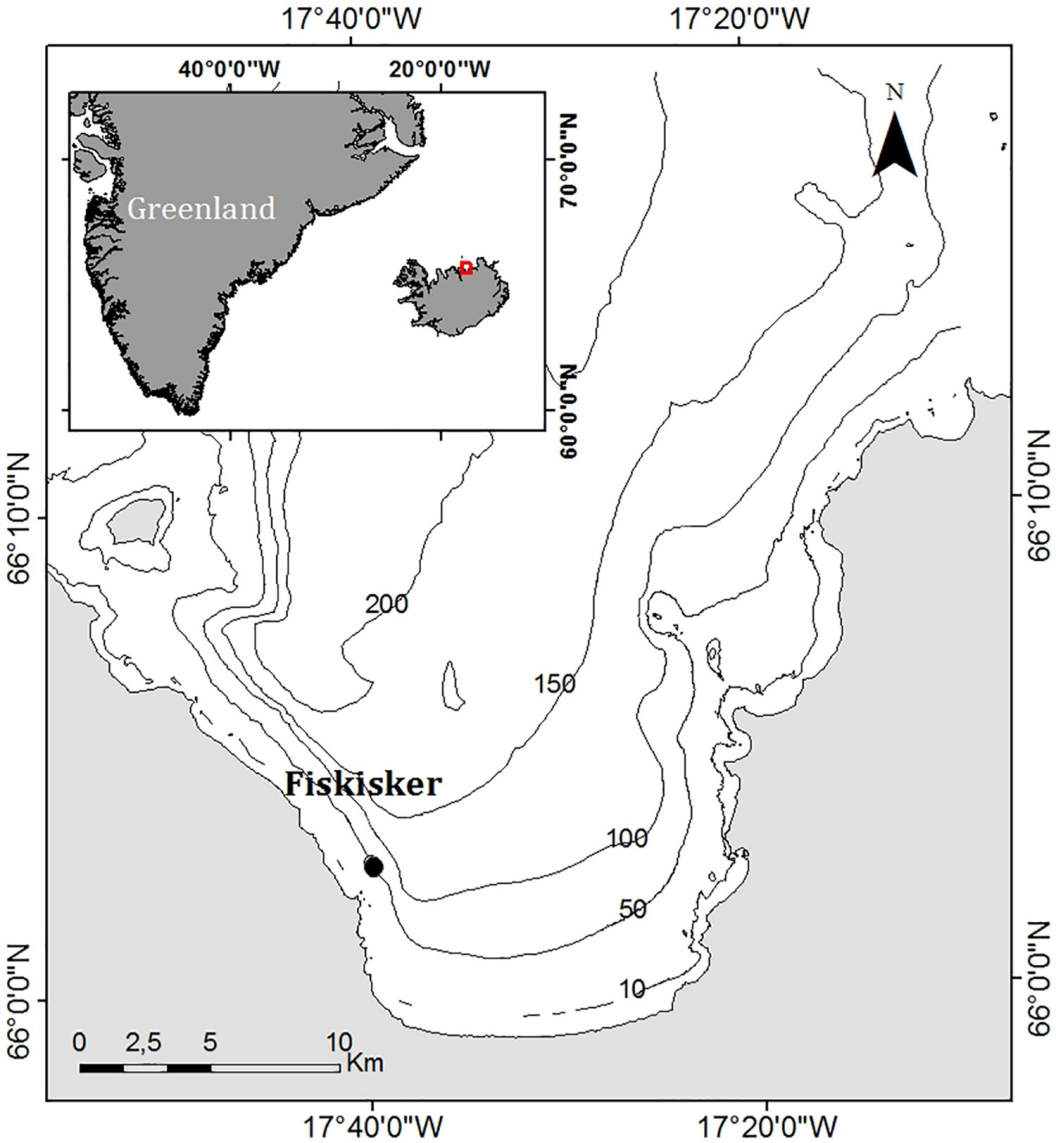
The study area in Skjálfandi bay, NE Iceland. The black circle represent the location of the EAR recording unit at Fiskisker (66°03’N, 17°40’W). Depth contours are in meters. Source (1) Hydrographic Department of the Icelandic Coast Guard, 2012, (2) National Land Survey of Iceland, 2012 and (3) Esri, DeLorme Publishing Company, Inc. The map was created using ArcGIS_ software (version 10.1) by Esri. Adapted with permission from Magnúsdóttir *et al*. [37].

Humpback whale songs were collected from January 26^th^ to March 12^th^, 2011, in Skjálfandi Bay, Northeast Iceland using the methods described in Magnúsdóttir *et al*. [31, 37]. The recordings were made with a single bottom-moored ecological acoustic recorder (EAR) located approximately 62 m in depth on the slope Fiskisker (66°03’N, 17°40’W) (Fig 2). The EAR is a microprocessor-based autonomous recorder containing a Sensor Technology SQ26-01 hydrophone that has a response sensitivity of -193.5 dB (±1.5 dB) and is flat from 1Hz to 28 kHz [54]. EARs are described in detail in Magnúsdóttir *et al*. [31] and Lammers *et al*. [54]. EAR detection ranges are estimated to be from 12 to 28 km for humpback whale signals below 1 kHz. This is based on a minimum (171 dB) and maximum (189 dB) source levels and assumes spherical spreading. The EAR was set to record for 10 minute intervals every 15 minutes at a sampling rate of 16 kHz to capture the fundamental range of humpback whale songs (approximately 8–8000 Hz) [25, 28] for approximately 1.5 month period.

### Song detection

A frequency contour detection algorithm from the Ishmael 2.0 software package was applied to search for tonal signal frequencies in recordings that ranged from 100 to 1000 Hz [55, 56]. Detection thresholds were set to 0.25 seconds (FFT 0.2048 s., 75% overlap, Hamming window). Despite spanning only a part of the humpback whale tonal frequency range, the detector primarily detected humpback whale signals with minimal false positive detections. The signal detection rate per minute of effort for each day of recording was obtained using the Ishmael 2.0 software. All sound files with detections were checked for humpback whale songs.

**Fig 3:**
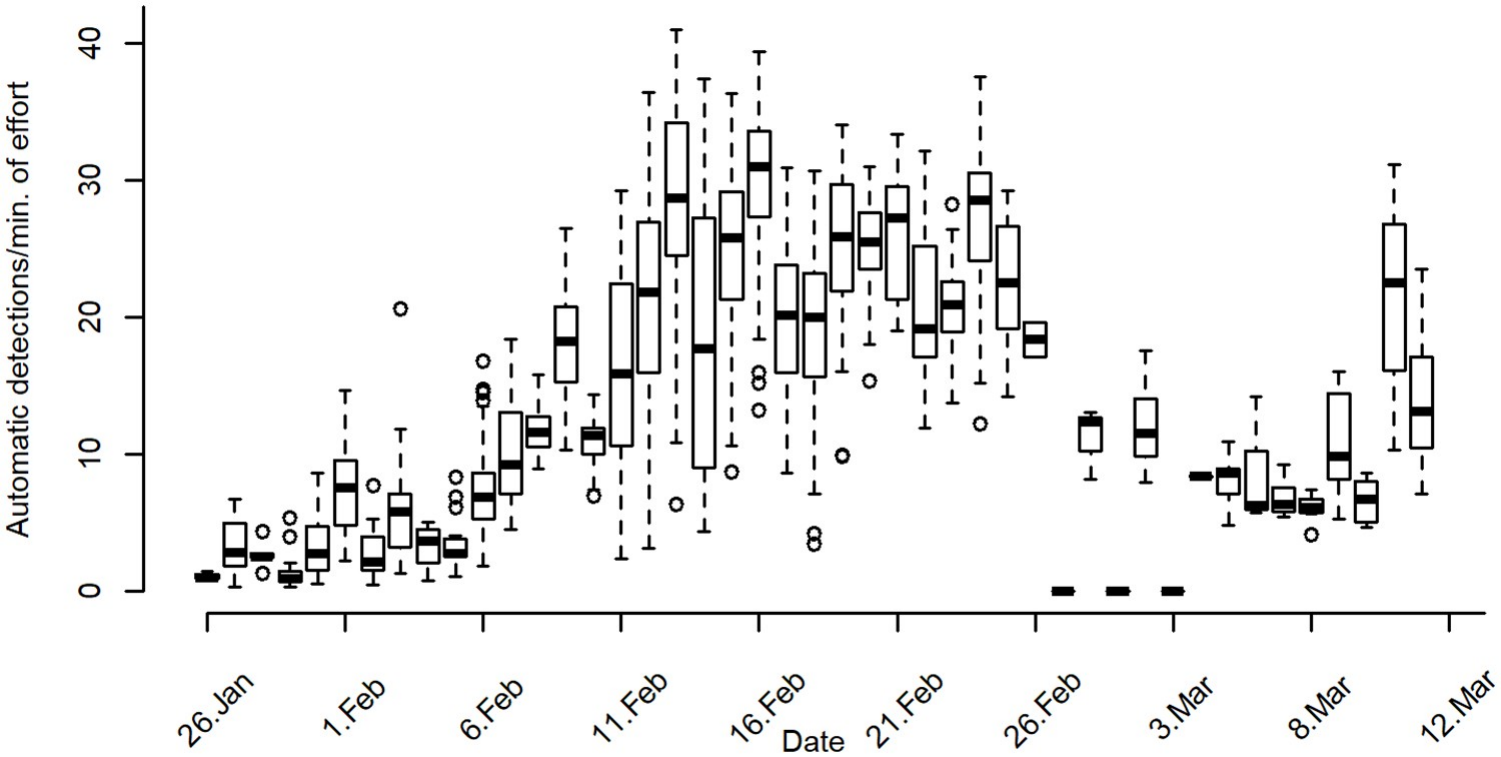
Song detections. Humpback whale song detection distribution per day of recording, i.e. during January 26^th^ to March 12^th^ 2011.

Several factors can affect the detection rate of an automatic detector, primarily the signal-to-noise ratio (SNR) of the target signal, but also the number of singers per sound file, the number of song units in the recorded phrases and the percentage of each recording which included the target sound (song units). A subset of 87 detected sound files with humpback whale songs was investigated to manually verify the number of singers in the sound files and to test for correlation between the minimum number of singers per sound file and the detection rate. The minimum number of singers per sound file was estimated by investigating if two or more phrases were sung at the same time. Song units that overlap in time cannot be produced by the same whale, therefore, these overlapping incidences provide the number of overlapping singers at a particular moment (see Figure A in S1 File for further clarification). More singers could be present, therefore, this method estimates only the minimum number of singers within the detection range of the recorders per sound file. The effect of SNR and the percentage of song in each recording on the detection rate was also measured to evaluate if that biased the estimate of the number of singers based on detections only. No visual observations occurred during this recording period in the winter, therefore no visual confirmations of the number of whales in the area during singing events could be made. The acoustic estimates can, however, be used to obtain an overview of when there were likely solo singers or dispersed singers in the area and when there were chorusing males within the detection range of the recorders. That was done by inspecting a boxplot (Fig 3) of the full dataset obtained from the automatic detector, without inspecting each sound file separately.

The signal-to-noise ratio (SNR) and audibility of the signals in each recording were manually inspected for recording quality. The sound files were graded and categorized as very poor, poor, medium, good, and excellent [57]. Files where all signal details from at least one individual were distinctly visible with high amplitude units and harmonics, i.e. where major parts of the song had a good SNR with a minimum of 10 dB above the background noise, were marked as good to excellent quality. Excellent quality sound files included larger proportion of high amplitude song units. A subset of good to excellent quality sound files was evaluated and the mean SNR per sound file was observed using a custom written algorithm in MATLAB version R2017b to verify the manual quality estimate. A total of 8 different song units of various intensity from each sound file from the subset were analyzed for SNR which gave a mean SNR for each of these sound files. Such measurements provide an overview of the different signal qualities in a recording which provides an aid to avoid the lesser quality recordings. This method allows for choosing recordings where some song units are weaker as long as the majority are stronger and clearer. The aim was to collect sound files which would result in a minimum of 10 dB total mean SNR for each recording period to allow for comparison between periods (see details in next section).

### Dataset creation from fractioned song recordings

Due to the long-term application and data storing limitations of the EAR recordings, the dataset was not continuous (i.e., each 10 minute recording contained 5 minute break intervals). The dataset did not contain many complete songs, therefore, complete songs could rarely be extracted directly from the recordings. To create a dataset for a group of singers, four to six adjacent sound files of excellent quality were acquired from 16 different days throughout the course of the recording period. These 16 different days were grouped into four distinct periods of four days each. By subsetting the dataset into even periods (sets) we could avoid biasing the results towards group of singers for which we had a higher number of song sequences. This allowed for comparison of songs between the quarters to search for temporal changes in the songs. Division into fewer periods could result in too low resolution of the data and, thus, higher risk of averaging out possible changes while greater number of periods would include too small dataset each. To provide a credible comparison between the four periods the same number of days was required for each period. As previously explained, the mean SNR for the sound files chosen for each period would need to be of minimum 10 dB. Though most of the sound files included humpback whale songs it was not always possible to find many excellent quality sound files in a consecutive order. The number of excellent sound files also varied considerably between the four periods. It was, however, possible to extract at least four sets of consecutive high quality sound files from each period. Each day represented the same or similar set of singers with 4–6 adjacent recordings, resulting in 40–60 minutes of singing during a period of 60–70 minutes (due to 5 minutes break between the recordings). Each set of sound files could cover at least a single song cycle, which usually lasts for 30 minutes or more [30]. It is considered likely that each collection of adjacent sound files contains songs from the same individual or the same group of whales since only 5 minutes past between the adjacent sound files. Sound files separated by a minimum of 24 hours were considered more likely to contain a different set of singers since previous studies have shown a small likelihood of resampling the same individual with 24 hours passing by [49, 58]. As a result, this method created a dataset containing songs likely to have been produced by several different groups of singers, with each set comprising of 40–60 minutes of recordings from one given group of singers. This, additionally, allows for an inspection of variations within a set of the same individuals as well as variations from the total observed singers throughout the recording period.

Songs were inspected using the spectrographic view of the Raven Pro 1.4 program (Cornell Lab of Ornithology, Ithaca, NY) (Hanning window, 2048 DFT size, 50% overlap) and phrases were logged and labeled in a time sequence as they occurred on the spectrogram. Phrase names used in this dataset are consistent with the naming conventions used in the Magnúsdóttir *et al*. [31] study. In that study, units constructing song phrases were measured with statistical methods, categorized into groups, and named accordingly. Two observers (RL and EEM) verified the categorization of the phrases.

### Song delineation

The good to excellent sound files chosen for the previously described four different periods (sets) were delineated to obtain song patterns from each of these period. Authors often define varying start and end points for the song sequence, because of the variable and continuously evolving song sequence of humpback whales. Cholewiak *et al*. [59] advised against performing durational analyses on such variable data, and instead recommended that the methodology from established avian literature be applied to humpback whale songs. This involves focusing on phrase sequences and maintaining a consistent phrase description in the song analysis. Phrases are fundamental repeated patterns of 2 to over 20 units, and range from under 10 seconds to over 30 seconds in duration [27, 30], with a full song comprising of approximately 180 to 400 song units [60]. The complexity and variation of phrases depend both on the number of unit repetitions and as well on the composition of unit types and sub-phrases [27]. For this study, phrase delineation was considered to be the most effective and stable element for song structure analyses.

A new theme is initiated with a new type of phrase since themes are composed of repeated phrases. Transitional phrases are often observed between two phrase types, when one theme ends and another theme starts. These phrases combine features of both the preceding and succeeding themes [30]. Transitional phrases were found between almost all themes in the songs of the present study, therefore, the preceding and succeeding phrases could be assigned with strong certainty to the same singer. The advantage here is that transitional phrases unmistakably represent a correct order of themes from a singer when songs are overlapped by multiple singers. During several incidences transitions could be identified without a transitional phrase between themes, these were, however, from recordings of solo singers. To obtain the correct sequences of phrases, and ultimately themes, when singers are not visually identified, it is important to verify with as much certainty as possible that the themes within the delineated sequence belong to the same singer.

### The delineation protocol

Since the start and end of the sequence is not always clear, the start and end was determined visually by the observers as suggested by Cholewiak *et al*. [59]. The Markov analysis (explained in the following subchapter) revealed the most likely starting phrases (i.e. phrase 17 and 15) and ending phrases (i.e. phrases 4a and 4b) of the sequences, referred to as the “start label” and “end label”. Furthermore, if the same phrase reoccurred within a sequence, the sequence was considered terminated, with the subsequent phrase beginning a new sequence, e.g.:

1) [**15**-14a-13b-12-4b-**15**-14a] → [**15**-14a-13b-12-4b], [**15**-14a]

where each character (number or number and letter) represents a single theme and the hyphen indicates the transitioning event between the themes. Sequences are shown within the brackets. Since ‘15’ occurs twice within the first sequence, this sequence is terminated at the phrase ‘4b’ which is the phrase directly preceding the second incidence of ‘15’. This results in two shorter sequences where the first sequence constitutes a full song according to the protocol while the latter sequence is not a full sequence. If a sequence included the typical starting and ending phrases the sequences would be split up where these phrases met instead of following protocol 1), e.g.:

2) [13b-12-**4b-17**-14b-13a-13b-12] → [13b-12-**4b**], [**17**-14b-13a-13b-12]

Here, neither of the resulting sequences are full song sequences but represent a theme order which could be compared with other sequences.

### Analysis of phrase sequences

To account for the fractioned recordings, instead of continual recordings, a Markov transition analysis was applied to each of the four periods to estimate the most likely sequence of phrases belonging to a full song cycle. The Markov matrices calculate probabilities for each occurring transition, providing results that can be used to determine whether or not the phrase belongs to the same sequence. This is a common method used to interpret bird song organization and predict dependent behavioral states [61–64]. Where multiple singers were recorded singing simultaneously, the phrases from each singer were tracked manually if transitions between phrases were clearly visible on the spectrogram (see Figures D and E in S1 File). Delineation was terminated when there was too much overlap of similar phrases sung by different whales (see Figure F in S1 File).

A Fishers Exact test was used to estimate the consistency of phrase transitions between periods to investigate the progression of songs in the area throughout the recording season. The null hypothesis was that each phrase would transition proportionally the same to the same phrases in all periods. To test the hypothesis for each phrase, a contingency table was created for each phrase type, with table rows representing the periods and columns representing each phrase of which the phrase of interest was transitioning to. A *P*-value was calculated for each contingency table and assessed. If *P* > 0.05 then the null hypothesis was not rejected and it could be stated that the phrase of interest transitioned consistently to the same phrase or phrases in all periods. In other words, the transitions were non-random between periods. However, if *P* < 0.05 then the null hypothesis could be rejected and it could be stated that the phrase of interest did not transition consistently to the same phrase or phrases between the four periods, i.e. transitions occurred randomly between periods. Phrases that transitioned inconsistently to different phrases were identified as shifting phrases while phrases transitioning more consistently were identified as static phrases.

A quantitative method based on the Levenshtein distance (LD) technique [65, 66] was used to evaluate the similarity of observed transitional sequences between periods, i.e. sequences with a minimum number of four transition phrases that were extracted directly from the recordings (Figures D and E in S1 File). This method was applied to exclude small sequence fragments which are unlikely representatives of song sequences.

The LD calculates the minimum number of changes, i.e. insertions, deletions and substitutions, needed to transform one string of phrases into another [65–67]. A representative string, called the *Set median* (SM), was found for each of the four periods (sets) and used to compare sequence similarity between periods [52, 66–68]. Each string of phrases within a given period was compared to all other strings within that period. The SM was the string of phrases with the smallest summed LD compared to all other strings in the set. To ensure that the SM was the best representation of each period, a set of hypothetical medians, called *Kohonen medians* (KM) [65–67], were created to find if a smaller summed LD score could be obtained. The KM is created by systematically substituting each phrase in the sequence with all possible phrases found within the set (period). If the KM had a smaller summed LD than the SM, the KM would be used instead of the SM as a representative sequence for that period.

To investigate the similarity between the representative strings (SM or KM) for each period a Levenshtein distance similarity index (LSI) was used. The LSI normalizes the LD score against the longest string [52, 65, 66, 69]. The incorporation of the string length into the analysis allows the LD scores to be standardized so that the length of strings being compared does not increase the difference between the two strings. This enabled the difference in phrase types and phrase order to be the primary determinant of string differences. The LSI produced a proportion of similarity that ranged from 0 (0%) for no similarity to 1 (100%) for complete similarity between a pair of strings. The resulting LSI scores formed a matrix of LSI similarity which was converted to dissimilarity by subtracting each score from 1.

Using the dissimilarity matrix and the statistical program R (version 3.1.2), the representative sequences (SM/KM) for each period were hierarchically clustered. The single-linkage clustering (nearest neighbor clustering) method was employed to place the most similar sequences together. These sequences were then successively linked to other sequences/clusters of sequences [66]. This method analyzed how similar the representative sequences were between periods, allowing for evaluation of song sequence progression. Additionally, the same clustering analysis was applied on all the extracted sequences (song fractions), with a minimum of four different themes, from each period (set) to investigate the variation of the sequences extracted (song fractions) from each period.

The presence and sharing of phrases within the songs and between periods was inspected using Dice’s similarity index [70]. Note that this analysis does not consider the sequential characteristics of the songs. Dice’s coincidence index was originally designed as a measure of the amount of association between two species [71]. Here, the index is used as a measure of phrase sharing (a method that was previously used by Garland *et al*. [70]) between the four defined periods:

SI=2A/(B+C) (1)

where SI is the song phrase similarity between population pairs, *A* is the number of shared phrases, *B* is the total number of phrases present in population-1 (e.g. period-1), and *C* is the total number of phrases present in population-2 (e.g. period-2).

## Results

### Humpback whale winter singing activity in the subarctic

During the 46 day recording period, from January 26^th^ to March 12^th^, 2011, songs were detected in 42 days (91.3%) in 1268 different sound files (10 minutes each). Songs could not be confirmed during only 4 days out of the 46 (8.7%). The examination on the random subset of sound files with humpback whale songs showed that the SNR levels per sound file, percentage of sound file with song units and the number of singers per sound file correlated positively with the detection rate (Figure B in S1 File). That needs to be taken into account when evaluating whether chorusing whales or solo singers were present when examining the total detection trend throughout the recording period. The investigation of the random subset showed, however, that sound files with more than 15 detections per minute of effort always included two or more singers ‘chorusing’ while sound files containing more than 30 detections per minute of effort included at least four to five singers chorusing (Figures B (A) and C in S1 File). Detection rate below 10 detections per minute of effort were more likely to include solo singers. However, singers could sometimes be more than one despite of low detection rate when the SNR was low or only a small percentage of the sound file included songs. High rates of detection were captured every day from February 9^th^ to February 26^th^ 2011 indicating a more frequent chorusing of multiple whales during that period (Fig 3). Humpback whale songs were detected until mid-March when recordings ended. The average signal-to-noise ratio (SNR) varied between periods with the lowest SNR values during the first period, the highest during the third period and a slight decrease in the SNR values during the last period (4^th^) based on the random subsample of the delineated sound files. The average received signal level and detection rate from the subsample showed the same trend throughout the total recording period (Table D) indicating that the whales were more often recorded closer to the recording units as the recording period progressed and possibly increased in number.

**Fig 4:**
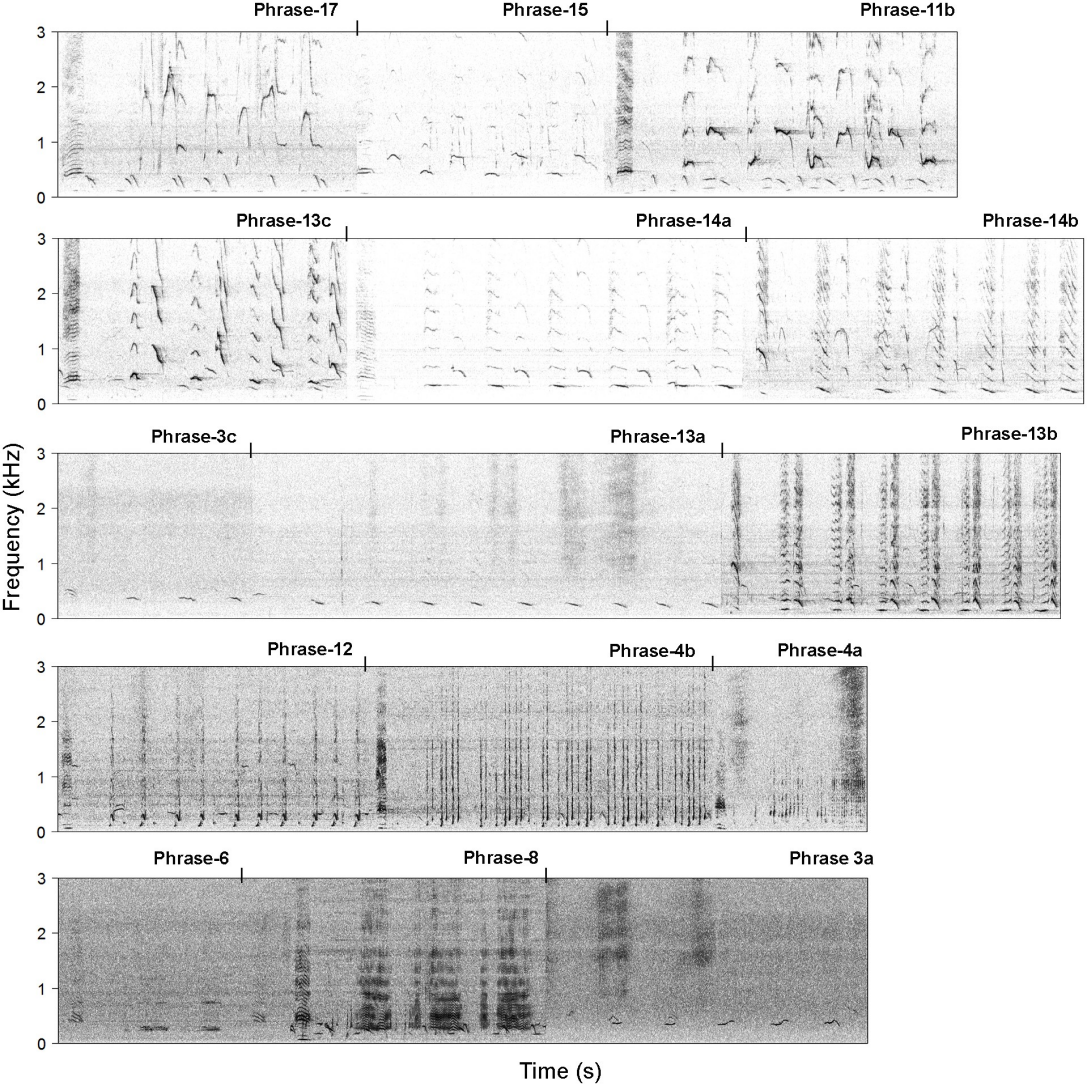
The observed phrases from 2011. Spectrographic representations of the observed phrases from the complete recording period. The spectrograms were generated using Fast Fourier Transformation (FFT) (size 2048 Hanning window) with a frequency resolution of 7.8 Hz and a 95% overlap. The vertical, black lines indicate the division between the phrases. Audio is provided in S1 Acoustic File.

In total, 70 medium to high quality 10-minute sound files were used for song analysis during the four defined periods (Table 1). This resulted in approximately 11:20:00 h of analyzed song files. From these recordings a total of 1683 phrases were logged and identified, resulting in 15 different song phrases (Fig 4), and consequently 15 theme types from the whole period. In total of 281 phrase sequences (song parts) were extracted from the dataset. Of these, 12 full songs were captured which fitted within 10-minute sound files. Sequence of themes, represented by phrases, would be considered a “full song” when a phrase type reoccurred in the sequence (Table B in S1 File). The song would end on the theme occurring before the first theme type re-occurred in the sequence. The average number of different themes in each full song which fitted within 10 minutes were 5.7 (SD = ±1.7), ranging between 3–9 different themes. When these full sequences were delineated according to the delineation protocol, where themes 17 and 15 were assigned as starting themes and themes 4b and 4a terminal themes, the remaining full sequences from these original 12 sequences were 7 and the average number of themes was 4.8 (SD = ±1.7), ranging between 3–8 different themes (Table B in S1 File). Two rare phrases were detected in the dataset which had not yet been assigned to the 2011 dataset in the previous Magnúsdóttir *et al*. [31] study where the same recordings were used. Because the present study collected a larger sample size, rare phrases were more likely to be observed. These two phrases (phrase-3a and phrase-6) had previously been observed during the winter seasons of 2009 and 2010 [31].

**Table 1:** A summary of the analyzed dataset.

Period	Dates	Time	No. sound files	Estimated no. singers (range)	No. song seq. analyzed for LD
1	27-Jan-11	22:30–23:15	4	3 (*1–3*)	3
	30-Jan-11	13:00–17:15	6	3 (*1–3*)	3
	31-Jan-11	04:15–05:00	4	3 (*3*)	8
	2-Feb-11	02:45–03:30	4	5 (*4–3*)	3
	*Total*		*18*	*11*	*17*
2	5-Feb-11	03:15–04:00	4	2 (*2*)	4
	7-Feb-11	05:45–06:30	4	3 (*2–3*)	3
	9-Feb-11	19:30–23:15	4	4 (*4*)	2
	10-Feb-11	00:00–00:45	4	2 (*2*)	7
	*Total*		*16*	*9*	*16*
3	18-Feb-11	21:00–22:15	6	4 (*3–4*)	6
	20-Feb-11	00:45–01:30	4	4 (*3–4*)	6
	24-Feb-11	17:45–18:30	4	3 (*1–3*)	7
	25-Feb-11	06:15–07:00	4	4 (3–4)	9
	*Total*		*18*	*12*	*28*
4	2-Mar-11	15:15–16:00	4	2 (*1–2*)	4
	8-Mar-11	01:30–02:15	4	1 (*1*)	4
	11-Mar-11	14:15–15:00	4	3 (*3*)	6
	12-Mar-11	06:30–07:15	4	1 (*1*)	2
	*Total*		*18*	*6*	*16*
Total song time examined		11h 20m			

The date, time, and number of consecutive song files analyzed per selected day including the number of confirmed singers per day. The range of the number of singers detected in the sound files per day is shown in parenthesis. A minimum of 24h intervals passed between detection dates to allow for the estimation of the total hypothetical number of singers. Only song sequences with a minimum of four different phrases were included in the Levenshtein distance analyses (LD).

Each phrase type observed in this present study represented a theme, where the phrase was repeated several times before transitioning into a new theme, most commonly via a transitional phrase. A total of 438 transitional phrases were obtained from the dataset (26% of the observed phrases) and used in the Markov analysis. A total of 77 transitional sequences were used in the Levenshtein distance (LD) analysis (Table 1), with a minimum of four phrase transitions required for analysis.

**Fig 5:**
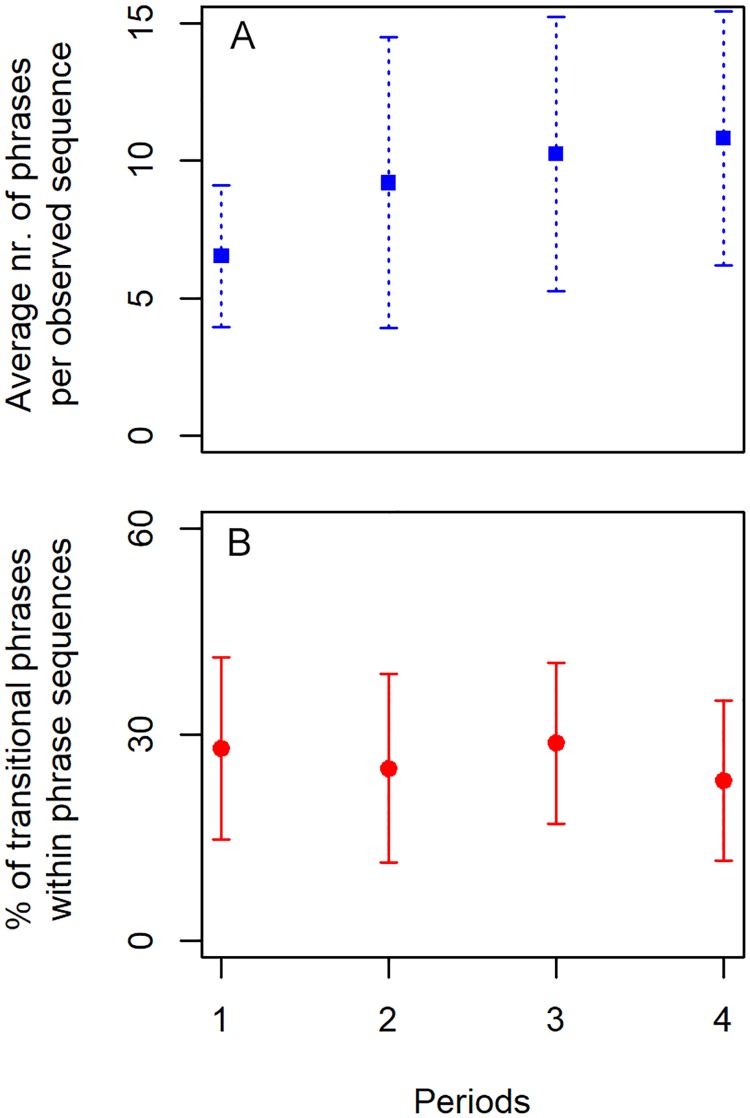
Occurrence of phrases and transitional phrases. A) An increase in the length of the phrase sequences was observed when including all repeated phrases within the 10 minute recordings. Sequences with fewer than four phrases were not included in this dataset. B) The percentage of transitional phrases within the observed phrase sequences from period-1 to -4.

The captured sequences including repeated phrases ranged up to 22 phrases within the 10 minute sound files. When only including sequences with a minimum of four phrases, the average length of the captured phrase sequences was 8.9 phrases (± 4.6) per observed sequence. The total length of the observed phrase sequences increased from period-1 to period-4 (Tukey’s differences of mean = 4.3 increase in no. phrases, F_3,163_ = 8.7, *P*<0.001) while the percentage of transitional phrases within the phrase sequences decreased from period-1 to -4, however, this change was not significant on the *P*-level (Tukey’s differences of mean = 4.6% decrease, F_3,163_ = 1.5, *P* = 0.2) (Fig 5).

Multiple singers, i.e. ≥2 singers, were detected on 77.6% of the analyzed sound files where the average number of singers per sound file was 2.6 (±1.1). Singers were rarely synchronized when chorusing. However, the singers usually conformed to the same song, but not necessarily to all the same components of the songs.

### Phrase development within songs and periods

**Fig 6:**
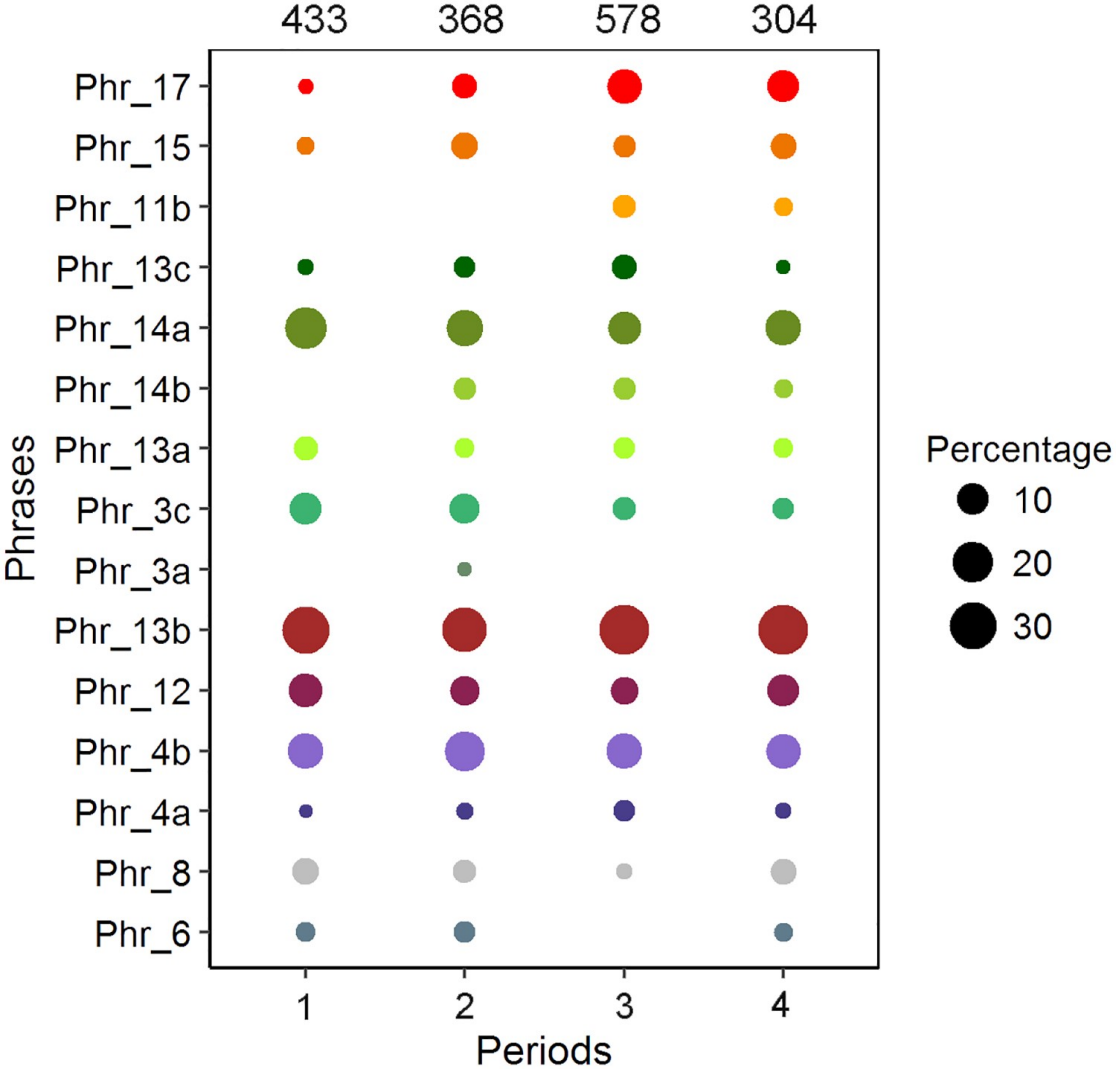
Percentage of phrase occurrence. Percentage of phrase occurrence within the songs per period. The size of the filled circles represents the prevalence of the phrases during each period, i.e. the larger circles represent greater prevalence of each particular phrase. The values above each period column represents the phrase sample size for each period (n).

The observed phrases could be categorized as static phrases or shifting phrases. The shifting phrases were less common than the static phrases and transitioned less consistently to certain phrases while the static phrases were included in the majority of the observed songs (Fig 6; Figure G in S1 File) and transitioned quite consistently to a particular phrase type for most singers (Table 2). Some of the rare phrases increased in occurrence as the period progressed, others gradually decrease while others remained rare in all periods (Fig 6). It should be taken into consideration that the lower average SNR from the first period (Table D and Figure H in S1 File) may have affected the detection of high frequency phrases (such as 17, 15 and 11b). The number of observed phrases increased from the first and to the second period where a total of 11 different phrases were observed during period-1, 14 different phrases during period-2, 13 during period-3 and 14 during period-4.

**Table 2:** Consistency of transitions of each phrase type between periods.

Phrase type	Occurrence during transitions	Difference in transitions between periods (*P* -value)
17	36	<0.001*
15	23	0.88
11b	10	1.00
13c	15	0.01*
14a	84	0.06
14b	11	0.29
13a	17	0.65
3c	34	0.07
13b	112	1.00
12	84	1.00
4b	23	0.34
4a	5	1.00
8	7	1.00
6	3	1.00
3a	1	1.00

Results from the Fishers Exact test indicating whether a single phrase transitioned consistently (*P*>0.05) or inconsistently (*P*<0.05) to other phrases throughout the course of the recording period, i.e. from period-1 to period-4. The asterisk indicates the phrases that were significantly inconsistent in their transitions to the next phrase across the four periods. The ‘Occurrence during transitions’ column indicates how often each phrase was included in a transition within the Markov matrices.

Dice’s similarity index (DSI) showed a high similarity of phrase composition between all periods (88%– 97% similarity). Though small, the similarity between periods decreased from the first and to the last period where the greatest difference in phrase usage was between the first and the last two periods, i.e. 88% similarity between periods 1 and 3 and 89% similarity between periods 1 and 4.

**Fig 7:**
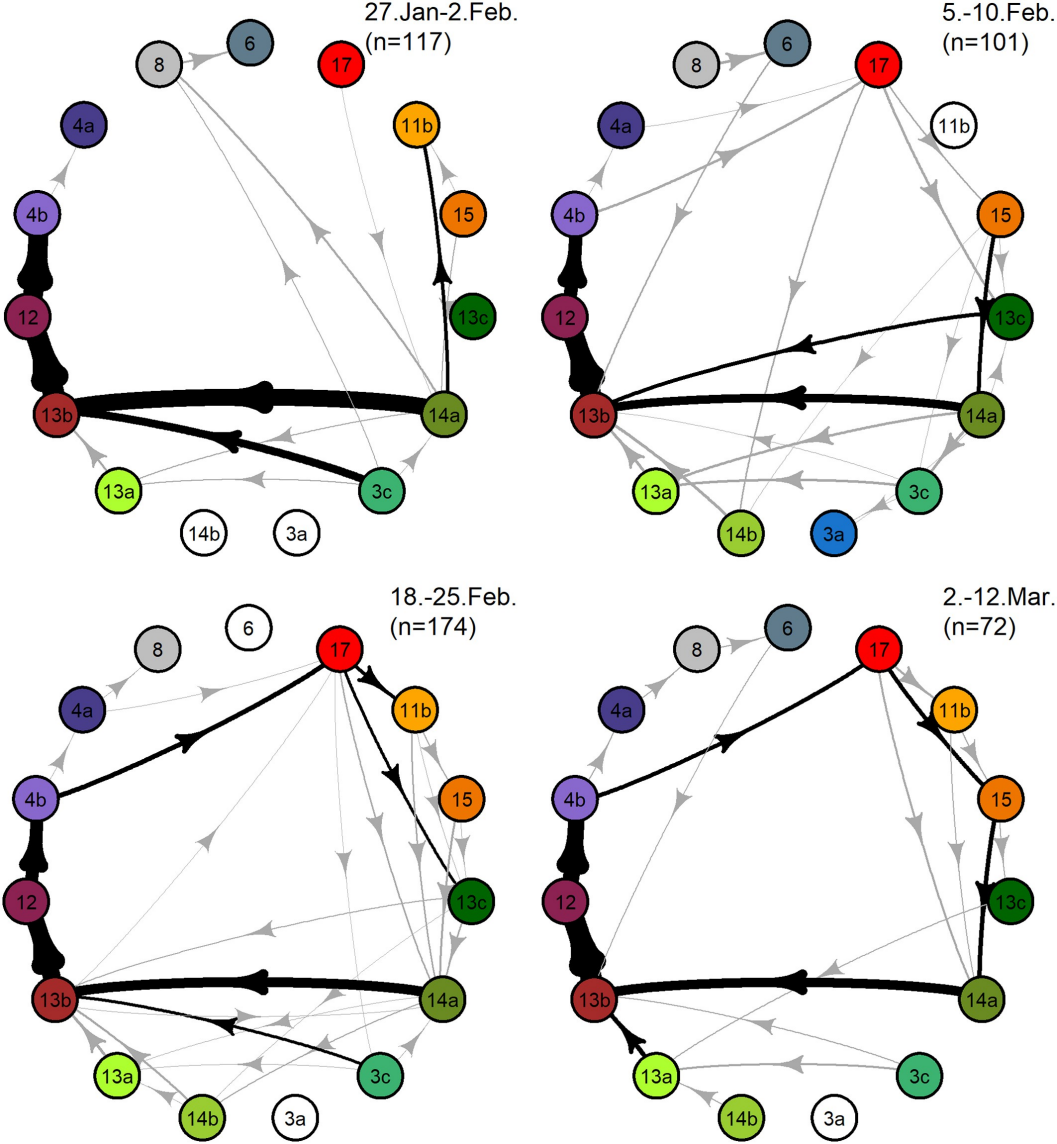
A diagram of phrase transitions. A diagram based on Markov transition matrix of song sequences during each period. Each circle in the The table summarizes the most likely sequences per period according to the diagram represents a phrase type. The colored circles are phrases which were Markov matrices. The set median sequences from each period are shown observed during each particular period while the white phrases were not observed during that particular period, only in other periods. The data points (n) represent the number of analyzed transitions observed during each period. Black lines represent transitions occurring a minimum of 5% of the time while gray lines represent transitions occurring less than 5% of the time. Single transition events are represented with thin light gray lines. The line thickness indicates the prevalence and frequency of the transitions observed, i.e. thicker lines indicate more common transitions.

The Markov matrices revealed each period’s fundamental phrase sequences and their consistency within the songs (Fig 7; Table 3). The variation within the song sequences, in terms of transitional variation, increased notably during period-2 and-3 but decreased again during the last (4th) period (Fig 7). However, the Fishers Exact test showed very small variations in transition occurrence between the four periods, with most phrases transitioning rather consistently to certain phrases during the course of the recording period (Table 2). The less common phrases were rarely observed during transition events (such as phrase-6, -8, -4a, and -3a) and did, thus, not affect the results of the Fishers Exact test (Table 2). Three of the most common phrases, i.e. phrase-13b, 12 and 4b, transitioned predictably to certain phrases in all periods and thus represented static themes. Four shifting phrases, i.e. phrase-17, -13c, -14a, and -3c, which represented shifting themes, contributed to the greater variation in transitions between periods (Fig 7; Table 2; Table C in S1 File).

**Table 3:** Extracted song sequences using Markov matrices and set median analysis.

Period	Common Markov sequences	Set median sequences
1	a) 14a-3c-13b-12-4b	14a-13b-12-4b
	b) 14a-13b-12-4b	
2	a) 14a-13b-12-4b	14a-13b-12-4b
	b) 15-14a-13b-12-4b	
	c) 13c-13b-12-4b	
3	a) 17-11b-14a-13b-12-4b	17-14a-13b-12-4b
	b) 17-13c-14a-13b-12-4b	
	c) 15-14a-13b-12-4b	
	d) 14b-13b-12-4b	
	e) 13a-13b-12-4b	
4	a) 14a-13b-12-4b	14a-13b-12-4b
	b) 17-15-14a-13b-12-4b	
	c) 13a-13b-12-4b	

The table summarizes the most likely sequences per period according to the Markov matrices. The set median sequences from each period are shown.

### Phrase sequence progression

A fundamental sequence of static themes, i.e. [13b-12-4b], occurred at the end of the majority of extracted sequences and was also the most common Markov sequence (Fig 7; Table 3). Theme sequences occurring before this fundamental sequence varied within the songs between periods (Table 3; Figure G in S1 File). The designated starting themes or themes early in the songs (17, 15, 11b and 13c) included high frequency units which had rather varying contours between singers, these were followed by themes which gradually lowered in frequency (14a, 14b, 3c, 13a and 13b) and transitioned into themes composed of repeated short upcall units (12, 4b and 4a) which sometimes were followed by themes made from low frequency impulsive units (8 and 6) (Fig 4). The set median sequences obtained from the sequences extracted directly from the recordings were consistent with the most common sequences produced by the Markov matrices (Table 3). The hypothetical Kohonen set median sequences did not produce better representatives of sequence sets for any period, thus, the set medians obtained from the true data were the best representatives for all periods.

The set median sequences for period-1,-2 and -4 were the same, i.e. [14a-13b-12-4b]. The set median sequence from period 3 was almost the same only phrase-17 was also included, i.e. [17-14a-13b-12-4b]. Since the set median sequences from all periods displayed 80–100% similarity between each other according to the LSI analysis, the songs analyzed from these four periods could be considered a single cluster of songs [66]. However, the variance in the usage of shifting phrases was noticable, particularly during the latter periods (3 and 4) resulting in a greater variance in sequences being sung (Table 3; Table A and Figure G in S1 File) as the number of singers likely increased (Fig 3; Table D in S1 File). The full song sequences captured within the 10-minute sound files and presented in Table B in S1 File show that there was an individual variance present in the use of themes, in theme order and in the repetition of phrases. That would evidently add to the variance of sequences with an increased number of singers in the area. According to the measurements done on a subset of delineated sound files (Figure B in S1 File) it was clear that the SNR and the number of singers affected the detection rate. That indicatest that fewer singers were in the study area and mostly singing further away from the recorders in late January/early February (period-1) compared to the latter periods. Therefore, some of the high frequency phrases, such as phrase-17, may not have been detected on some of the analysed sound files despite that a large proportion of the song units in each analysed sound files were equal to or exceeded the 10 dB SNR.

## Discussion

This study provides, to the best of our knowledge, the first description of the structure and progression of humpback whale songs from a subarctic feeding ground during the breeding months of winter. The findings demonstrated a characteristic song type for this region sung by the majority of the singers recorded during the study period. A fundamental theme sequence was included in the vast majority of the observed songs. Other themes were also included consistently, though not as rigidly, as these fundamental themes. When implemented in the songs, despite being predominant or rare, most themes were found to be in a similar pattern or place in the songs. The transition from one theme to the next was relatively consistent throughout the recording period for the majority of the themes. Transitions from only two phrases were significantly different across periods, i.e. from phrases 17 (*P*<0.001) and 13c (*P* = 0.01), and accordingly they were identified as shifting phrases and shifting themes. Two other common phrases were categorized as shifting phrases even though their transitions were not significantly differently between periods, i.e. phrases 14a (*P* = 0.06) and 3c (*P* = 0.07). However, their transitions were less consistent compared to other common static phrases and behaved, in that sense, more similar to shifting phrases. The lack of predictability, or entropy, of the theme transitions increased close to and around the middle of the period according to the Markov analysis but lowered again and returned back into fewer types of transitions close to the end of the recording period. Some variation in the rate of occurrence of themes was observed throughout the period as shown in Fig 6. Song progression was identified during the course of the recording period. These changes were primarily recognized in the change of theme use and in the increased number of phrases within the captured sequences as the period progressed.

### Characteristics of subarctic winter songs

The song characteristics observed in this study closely resemble reported songs quantified from low latitude breeding grounds. In particular, songs recorded in this subarctic feeding region displayed a sophisticated, hierarchical structure and a fundamental theme order shared by almost all of the observed singers. Themes were generally sung in the same order by all the detected singers but not in synchrony which has shown to be typical for humpback whale singers on breeding grounds [30, 72]. The observed songs were comprised of 14 unique song units, as discussed in the previous publication by Magnúsdóttir *et al*. [31]. These units subsequently made up 15 phrase types which were observed to combine and form 15 different themes. Of these 15 themes only five occurred regularly with other themes being less common. Some of the less common themes likely represented a variation of some of the more common themes. The full sequences showed individual variation in the usage of themes in two aligned song sessions. Therefore, it is likely that individuals sing different variations of the same theme or similar theme which can be interpreted as two (or more) separate themes by human observers. That must be taken into consideration when evaluating song variation within and between time and locations. Since only a small proportion of the analyzed sequences were actually full songs it is not clear how many themes on average constituted these songs. However, the full songs captured within the 10-minute recordings ranged from 3–9 different themes where the average number of different themes was 5.7.

Many song sequences exceeded the 10 minute recording, thus falling within the previously published average song duration range, i.e. approximately 6–30 minutes [30, 72, 73]. Additionally, reviewing consecutive sound files provided evidence for song cycles that lasted 40–70 minutes. Transitional phrases represented a relatively large part of the observed songs. Transitional phrases are characterized as unusual and complicated, and generally increase the entropy, i.e. decrease the predictability, of songs [48, 60]. Therefore, songs with less entropy have increased predictability [60]. Payne *et al*. [48] reported that Hawaiian humpback songs were only 5% transitional phrases, with the proportion decreasing towards the end of the season. The majority of changes between themes occurred through the use of transitional phrases with a slight proportional change found towards the end of the season, indicating a possible decrease in the songs’ entropy. However, as the present study covered approximately 1.5 months of the breeding season, it may not have captured a true trend in decreasing entropy. In Payne *et al*. [48], songs were described as becoming more predictable and organized as transitional phrases were phased out. According to this definition, the subarctic Icelandic humpback whale songs recorded from the end of January until mid-March would be categorized as less stable.

The transitions observed in the present study between the more common phrases were actually found to be stable across all four periods with the clear exception of four shifting phrases, i.e. 17, 13c, 14a and 3c which represent shifting themes. This indicates that there is an apparent pattern of predictable phrases attributed to all periods which represent static themes. The consistent transition order found using Markovian sequencing showed that the sequences were fairly invariant and phrase transition reversals were rare throughout the entire recording period. In instances of varying phrase transitions between particular phrase types, a modified transition order would occur instead of the more common phrase transition. Such variation in theme orders are not uncommon in humpback whale songs and has for example been shown in songs from South Pacific breeding grounds [52, 73]. The greatest transition variations were found during periods that had the highest numbers of detections, i.e. period-2 and -3. The increased variations during these periods (Fig 7) were likely affected by both individual variation and a greater number of singers present in the area.

The most evident progression identified from the songs of this study was the gradual increase in occurrence of particular phrases, while other phrases were gradually removed from the songs as the period progressed. It is possible that the higher frequency phrases (e.g. phrase-17) were more common during the first two periods than shown by the results since the mean SNR and the mean received signal level for these periods was lower than during the two latter periods. The set median song sequences from all periods were clustered into a single group (>80% similarity) and 85–97% of the themes were shared between all periods suggesting that the songs analyzed from these four periods represent a single song type primarily constructed by three static themes and four shifting themes. In comparison to other studies, songs from six different breeding grounds in the South Pacific showed four different vocal clusters based on the LSI and DSI methods where similarity within clusters were minimum of 40% [66]. A limitation to this study is that full song sequences were not commonly extracted from the sound files due to the 10-minutes duration of each sound file. Therefore, the resulting delineated sequences sometimes showed a large variation since it varied what part of the sequence was recorded at each time. However, by comparing the transitions shown by the Markov matrices and the delineated song parts it is clear that the songs included constant static themes but varied due to the prevalence of various shifting themes. It is evident the songs had not matured into a stable song with a low entropy for this location though signs of decreasing entropy were detected during the last period in early to mid-March. High entropy in songs does not indicate that the purpose of singing is different than when the entropy has lowered, such variation in entropy has been reported from traditional breeding grounds [48]. It is possible that the variation in the usage and order of themes from this subarctic feeding ground is also affected by the shifting in individuals in the area at each time. This can, however, not be confirmed since the singers could not be identified visually. Nonetheless, the data indicated a varying number of singers in the area through the course of the study which supports this suggestion.

Since the results from the LSI and the DSI analysis suggest that the general song characteristics on this high-latitude feeding ground could be representative for this study area, the subarctic song would have the potentials to be transmitted to the North Atlantic breeding grounds. Studies have shown that humpback whales within an audible detection range of each other tend to conform to the same or similar songs and statements have been made that humpback whales within the same population do conform to the same song [47, 48, 74]. Therefore, we can assume that this song type was shared by other whales nearby and presumably by whales belonging to the same population. In Magnúsdóttir *et al*. [31], the repertoire of song units analyzed from this study area noticeably evolved during the course of three winter seasons while new phrases were being formed every year. Particular phrases from previous years were observed to be carried over to the next while other phrases were completely omitted after one season. These conformed changes over time indicate that humpback whale singers feeding in Icelandic waters, and likely other nearby central or eastern North Atlantic feeding grounds, share a repertoire of sounds. Such cultural development within and between years is continually shown on many traditional breeding grounds [e.g. 47, 49, 73, 75, 76] and likely occurs in this subarctic feeding ground.

### The strategy of the subarctic singers

The feeding grounds off Iceland’s coast are considered important habitats for North Atlantic humpback whales since a large proportion (approximately 80% of the North Atlantic population) are found to occur in Icelandic waters [77, 78]. A comprehensive photo-identification database is available for this region’s humpback whales identified in the summer but limited in the winter by the high subarctic location of this area. This particular location is characterized by polar nights (with darkness lasting up to 22 hours) and persistent adverse weather conditions throughout the winter season. Photo-identification matches of 50 humpback whale individuals were confirmed between summer and winter months of the same year in the Northeast coast of Iceland which indicated that those identified could be the same individuals recorded singing in the winter months [79].

In this study, humpback whale song vocalizations were detected almost every day (42 out of 46 days), from late January to the middle of March, 2011. Though song occurrences are mainly associated with traditional mating and breeding grounds of tropical low latitude aggregation areas, more findings confirm that singing is no longer an explicit behavior confined to such areas. Frequent reports of singing during migration already demonstrate the flexibility of singing behavior outside of regular breeding grounds [80–82] and songs have been recorded in numerous mid-to-high latitude feeding grounds, including feeding grounds located in the North Atlantic and North Pacific [38–41, 83]. Singing humpback whales have recently been tagged and observed during periods of active foraging behavior in the high latitude feeding grounds of the Antarctic during austral spring and fall [42, 43]. Findings by Stimpert *et al*. [42] demonstrated song production in close overlaps between singing and feeding behavior during periods of active dives at depths greater than 100 m. Their studies indicated that a trade-off strategy between foraging and mating behavior is highly applicable to the humpback whale species while on winter feeding grounds where spatial and temporal limitations are not as restrictive as previously assumed.

At this time, it is not certain whether the individuals remaining in the high subarctic waters are immature males rather than mature males singing throughout the winter breeding period. In Herman *et al*. [17], the majority of the singers during 10 winter breeding seasons in Hawaiian waters were reportedly sexually mature, with relatively few immature singers recorded. In their study, the participation of many male singers in the asynchronous singing chorus was interpreted as a lekking aggregation which could attract more females to the area with the heightened signal levels. The recordings from this present study included hours of singing, frequent chorusing of multiple singers, and long durations of complex song characteristics. Therefore, these recordings strongly suggest that sexually mature males were present in the area. Provided that females do also overwinter in the area, this aggregation of singing males may be a lek, thus, indicating a flexible and opportunistic mating strategy by humpback whales. Another alternative could be that the singing events in the subarctic serve as a preparation before reaching a specific breeding ground. Both alternatives support the notion that these singing events in the subarctic play a role in the humpback whales’ mating strategy.

This study supports Magnúsdóttir *et al*. [31] proposal that these subarctic feeding grounds may also be important for song exchange. Direct transmission and sharing can take place through mixing and communication between individuals sharing feeding grounds or during migration [43, 50]. The potential interaction and song exchange at the high latitude feeding grounds of Iceland could be a key driving force behind continued cultural transmission and song exchange between North Atlantic populations of humpback whales. Recordings that extend into the spring on the feeding ground and a comparison to songs sung in the same and following year on tropical breeding grounds are needed to better understand the reason for these subarctic winter songs and how they may contribute to this species mating strategy. Future investigations will quantitatively compare this study’s song recording with songs collected from known breeding grounds in the Cape Verde Islands and the Caribbean’s. Data collected from such a comparison would enable a confirmation of whether breeding ground songs are culturally transmitted in Icelandic waters.

On a global scale, perhaps humpback whales have always overwintered and partaken in late migrations from many or most mid to high latitude feeding grounds. Another plausible explanation could be that populations of humpback whales feeding in the high latitude areas of the North Atlantic may have adapted to a more delayed breeding period, e.g. as a response to food availability, compared to what has generally been reported for this species [12–15]. Therefore, the assumed peak breeding season could be a shoulder season for this population.

Equipment used in the past may have had limiting capabilities that hindered the ability to detect songs at high latitude feeding grounds. Though these considerations as well as the ecological contexts of an individual humpback whale complicate our understanding of the humpback whale behavior, it also presents a remarkable new perspective that demands further exploration.

## Supporting information

S1 Acoustic File A song phrases from the subarctic in 2011. The phrases are ordered as they occurred most often in the songs. A single representative phrase is provided for each theme and is the corresponding audio file for Fig 3.
(WAV)

S1 File Supplementary material document. The supplementary material includes supportive text, tables and figures for further clarification of the data set, the methodology and the results.
(PDF)
