# Supplementary material for: Subarctic singers: Humpback whale (Megaptera novaeangliae) song structure and progression from an Icelandic feeding ground during winter
Source: PLoS One. 2019 Jan 23;14(1):e0210057. doi: 10.1371/journal.pone.0210057 (PMC6343865; doi:10.1371/journal.pone.0210057)
Supplement: S1 File — The supplementary material includes supportive text, tables and figures for further clarification of the data set, the methodology and the results. (PDF) [file pone.0210057.s002.pdf]

Supplementary material for the submitted article:

Subarctic singers: Humpback whale (*Megaptera novaeangliae*) song structure and progression from an Icelandic feeding ground during winter

Edda E. Magnúsdóttir[1, 2], Rangyn Lim[1, 2]
[1]The University of Iceland's Research Center in Húsavík, 640 Húsavík, Iceland
[2]Department of Life and Environmental Sciences, University of Iceland, 101 Reykjavík, Iceland

Supplemental information on how the minimum number of singers was estimated

Estimating the minimum number of singers based on overlapping singing events within each observed recording.

With multiple singers chorusing in a single recording it is possible to evaluate the minimum number of singers that are singing at the same time within the detection range of the recorder. In this study, overlapping song units were used to estimate the minimum number of singers within each observed 10-minute recording. Within each of these recordings a minimum of two overlapping events were identified were the latter would verify the first estimate. There can be more singers within the recording than what the minimum estimate provides since the number of singers can only be evaluated with certainty when song units from different whales overlap in time. Therefore, the estimate only provides the minimum number of singers within the 10-minute time frame. That estimate can be useful when looking into the occurrence of peak singing events and when there are more or fewer than e.g. two singers in the area. The more singers that are overlapping their songs in time the more likely it is that more singers are within the study area then when few whales are overlapping. Examples of overlapping events where the minimum number of singers was estimated are shown in Fig A.

Importantly, the observers need to verify that some of the signals overlapping in time are not a harmonic component of a lower frequency signal used in the estimate. The contour of two different signals, used in the estimate, should be either 1) significantly different, 2) not aligned exactly in time or 3) the frequency gap between them should not equal the frequency of the lower signal. The purpose of the third criteria is to avoid treating harmonics of some signals as independent signals from another whale if criteria 1) and 2) are not met. The frequency range between two harmonics of a signal is the same as the frequency of the signal's fundamental component (the lowest tone in a harmonic signal). Signals become distorted with distance, therefore, observers should not use vague signals that are similar to other signals in the estimate.

The coloring of the song units in Fig A was done in Photoshop CC version 2014.2.0 using the magnetic lasso tool to track the contours of the signals and select them. Using this tool provides more accurate coloring of the signal contour instead of coloring by hand. Once a signal was selected a paint bucket tool was used to fill up the selected signal with color.

.

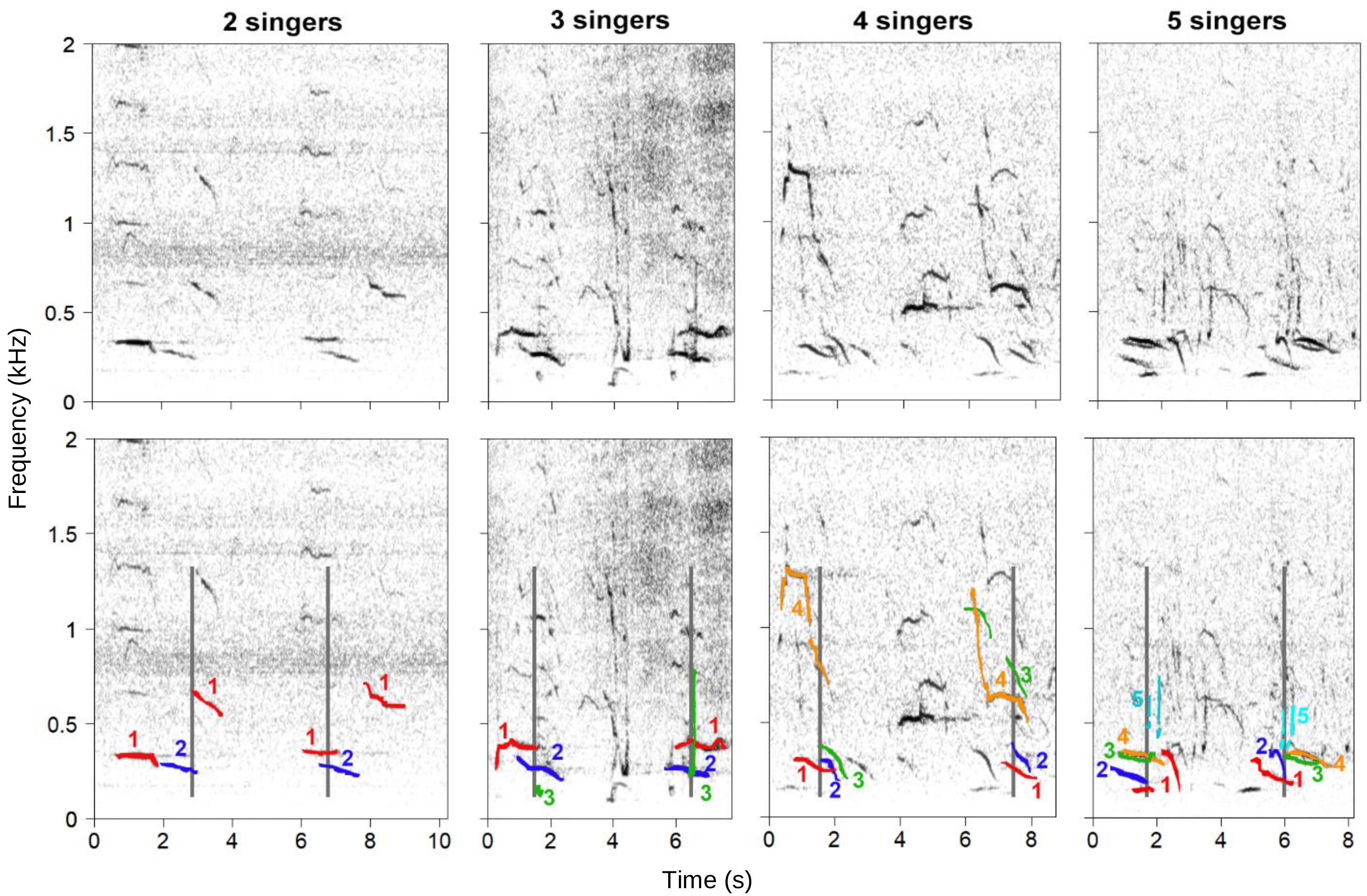

Fig A. Spectrograms of overlapping singers. The upper panel shows spectrograms of singers overlapping their songs in time. The lower panel shows the same spectrograms where each song unit, used in the overlapping estimate, is colored and labelled with a number representing each whale in the overlapping event. Two overlapping events are shown in each spectrogram. The vertical gray line shows where an overlap event occurred between the colored signals in time. Each spectrogram is independent from the other and were from various times of the study period. The colors and numbers are only to discriminate between singers during an overlapping event. In the first spectrograms where two singers overlap, a sub-phrase is identified from one whale. The sub-phrase included two song units that were labelled nr. 1 and identified with red color.

Supplemental material on humpback whale song unit detection rate

Estimating the influence of the 1) number of singers, 2) signal to noise ratio and 3) percentage of sound file with song units on the rate of song unit detections by the automatic detector

A subset of 87 sound files was randomly selected from the total 1268, 10-minute sound files containing humpback whale songs. The sound files constituting the subset were from the whole recording period. The resulting correlation between the number of singers per sound file and the detections per minute of effort per sound file is shown in Fig B (A) with clearer demonstration of that result using boxplot in Fig C. Additionally, the effect of the average signal-to-noise ratio (SNR) per sound file on the detection rate was tested including the effect of the proportion of song units during each 10-minute sound file on the detection rate. The proportion of song units within a sound file was estimated manually in Adobe Audition 2.0. Since the tested variables were not normally distributed the non-parametric Spearman's rank correlation was used on the data. There was a significant correlation between the detection rate and the tested variables as shown in Fig B (A-C) which demonstrates how these variables affected the detection rate.

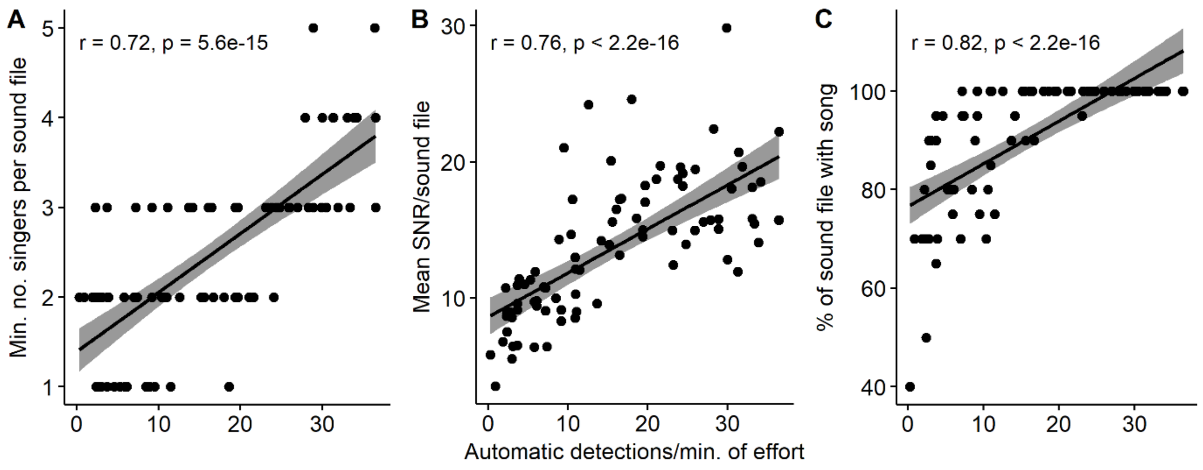

Fig B. Testing the effects of three different variables on the automatic detections of songs. All tested variables showed significant positive correlation with the detection rate by the automatic detector using the non-parametric Spearman's rank correlation (r =roh) indicating a significant effect on the resulting detection rate (per minute of effort) in each sound file. A) correlation between the minimum number of singers per sound file and the detection rate, B) correlation between the signal to noise ratio (SNR) per sound file and the detection rate, C) correlation between the % of each sound file which included song units and the detection rate.

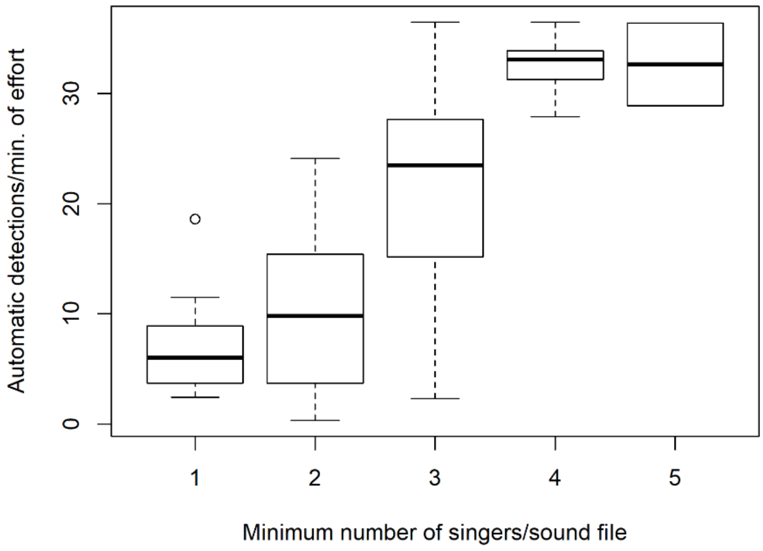

Fig C. Automatic detections of songs vs. the number of singers. A boxplot showing the distribution of automatic detections with increased number of singers per sound file. All examined sound files with more than 15 detections per minute of effort, apart from one, included two or more singers. If the detection rate exceeded 25 detections per minute of effort the sound files included at least three singers while sound files with more than 30 detections per minute of effort included 4 or more singers.

Supplemental information about song sequences and delineation

Usually, the delineated sequences were not full sequences since the recordings were set to record for 10 minutes every 5 minutes. A full 10 minute recording is provided in Fig D and the same recording with delineated phrases is provided in Fig E.

When chorusing whales are recorded the chorusing can prevent the observer from delineating songs from a spectrogram. This happens when chorusing whales are singing phrases which include song units of similar intesity (likely due to a similar distance from the hydrophone) and similar frequency, particularly when two or more whales are singing the same or similar phrases at the same time. When delineating songs qualitatively (since no autonomous methods are currently available) the observer needs to know where one phrase ends and another one starts. Transitional phrases assist the observer to know with good certanty what phrase type comes after another phrase, that can be useful when more than one singer is within a recording. However, such transitional phrases do not always occur or may be masked in a chorusing event. An example of a recording is provided in Fig F where phrases from at least four whales are overlapping. This one minute clip from a ten minute recording includes overlapping phrases of similar frequency and intensity. The overlapping of phrases in this recording was too intense for a human observer to know with certainty where one phrase is ending and another one is starting. Such parts of a recording can, thus, not be used and prevent the observers to delineate a full song or, as in this case, a full 10-minute recording.

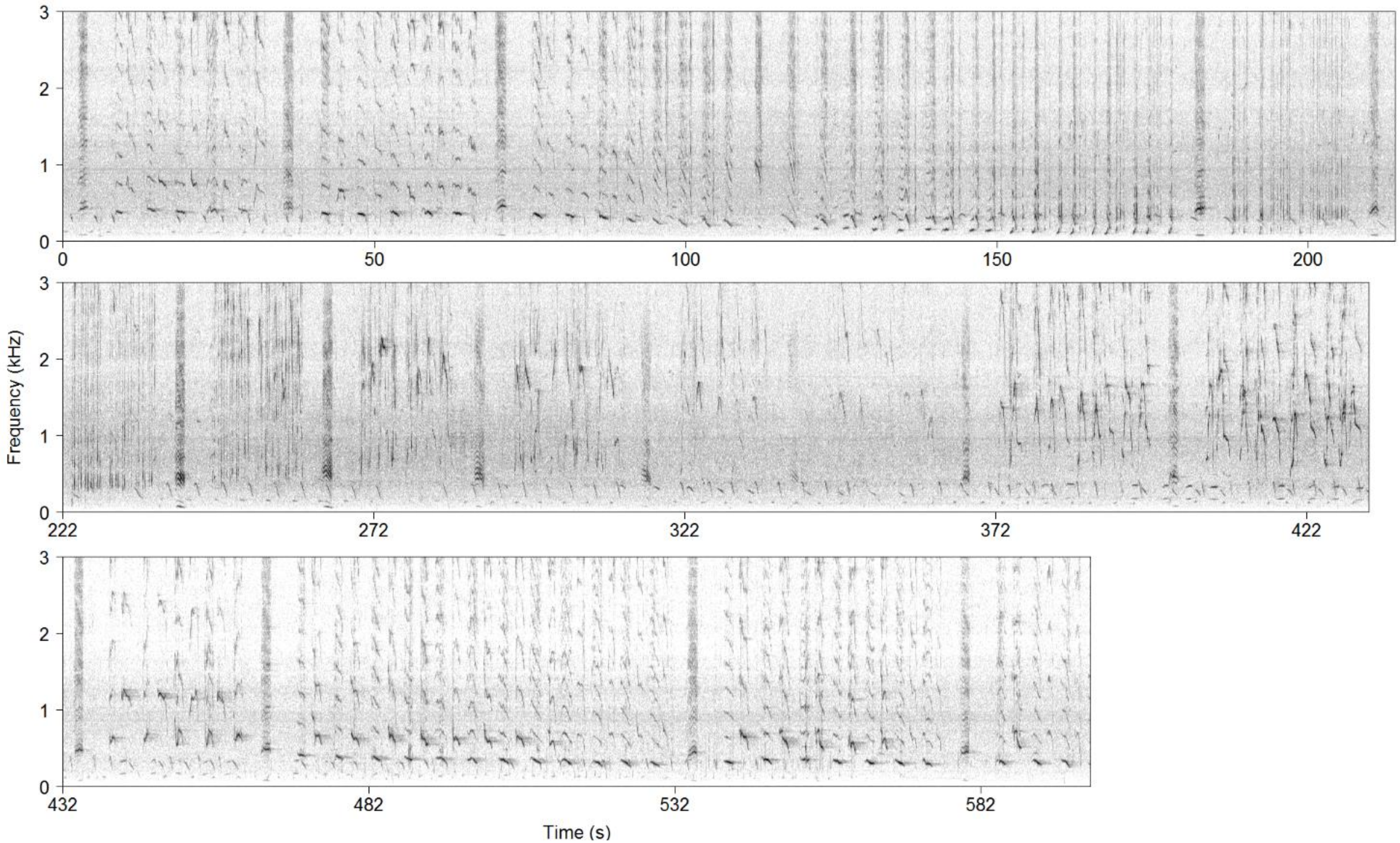

Fig D. A spectrographic view of a 10 minute song recording. A spectrographic view of a 10 minute recording showing phrase sequences from songs of at least 3 different singers . The whales are appear to be singing the same song but asynchronously. One of the singers is likely singing closer to the hydrophone than the other two since the song units of that singer's song are clearer on the spectrogram (with greater SNR). Such circumstances may make it easier for observers to delineate the songs found in the recording if there is a regular overlap of song units from different singers.

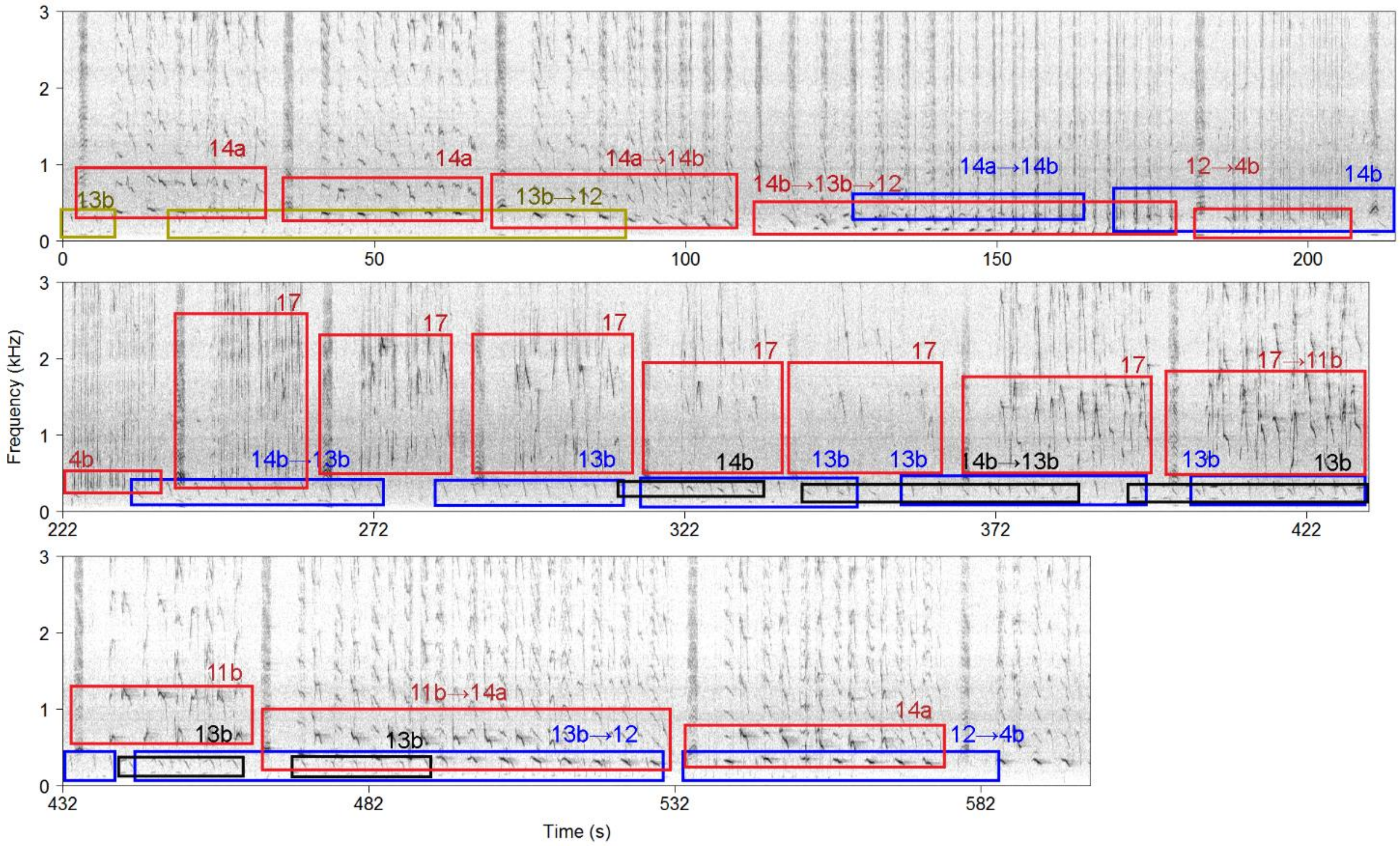

Fig E. A spectrographic view of a 10 minute song recording with delineated phrases. The same spectrogram as shown in Fig D with the song phrases now delineated. Each singer has an assigned color, with a single phrase identified using both a colored box and a corresponding colored phrase label (singer A = red, singer B = green, singer C = blue, singer D = black). Arrows between phrase labels indicate a transitional phrase. Singer B's signal attenuates at the beginning of the recording and because it is not certain if singer D is the same singer, a new label is assigned. At least 3 songs overlapped at time periods within the recording, indicating that a minimum of 3 singers were singing within the detection range of the EARs during this recording. A full song sequence captured from singer A, measured from phrase-14a to phrase-14a, lasted approximately 8 minutes and 50 seconds.

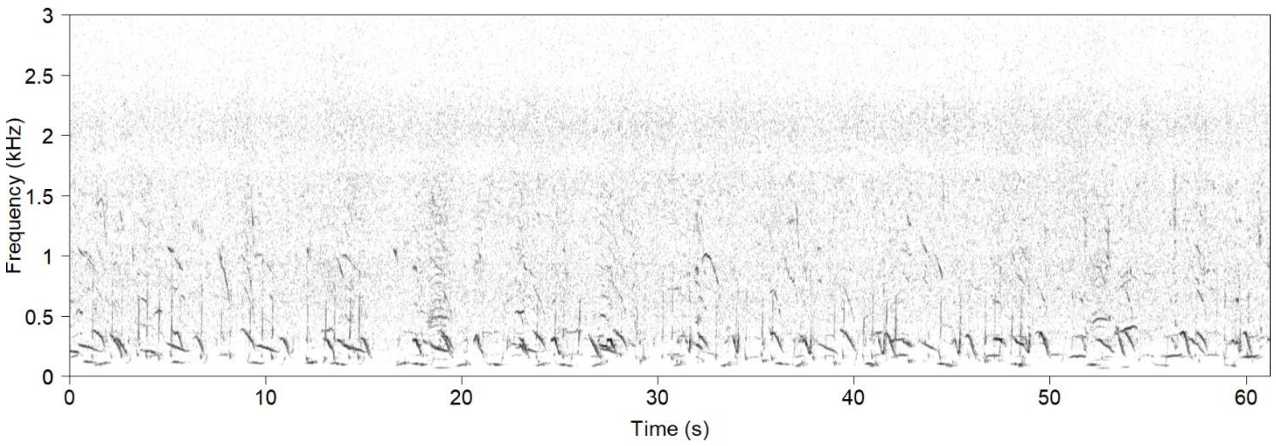

Fig F. A spectrographic view of songs from different humpback whales overlapping intensely. Different song units are easily identifiable from this spectrogram, also, a trained observer can recognize different phrases occurring in this recording. However, it is not clear where an individual whale starts and ends a phrase nor when that same whale starts singing a new phrase or repeating the previous one. That is due to either similar phrases sung at the same time resulting in a cluster of similar song units where it is impossible to identify what singers sang which phrase and units, or where louder units mask weaker song units, thus, making it unclear where the weaker phrase starts and/or ends.

The dendrograms in Fig G show the variation in sequences obtained from delineated recordings from each recording period. It is evident that the variation would likely be less if the delineated sequence had never been cut off, thus showing only full sequences and a true variation. The dendrograms in Fig G show, nonetheless, the predominant phrases in the sequences, i.e. “14a-13b-12-4b”, and provide a good overview of the variation in song sequences from the study period.

The assigned set medians (SM) from each period contained the predominant sequences found in the majority of the phrases, i.e. “14a-13b-12-4b”, however, with an increased number of different sequences and phrase types the summed distance of the set medians increased (Table A), indicating that with greater number of song sequence variants the less representative a single song sequence is for a given period. The mean Levenshtein Distance (LD) score of each SM for each period increased from the 1st period and to the 4th, also the number of song sequence groups (created with hierarchical clustering) increased, though not as substantially. Since the set medians were mostly the same in all the periods it is likely that only one song was sung in the study area during the recording period with a shared sequence within the majority of the songs. However, the variance in the usage of phrases appart from the static sequences was very high, particularly during the latter periods (3 and 4).

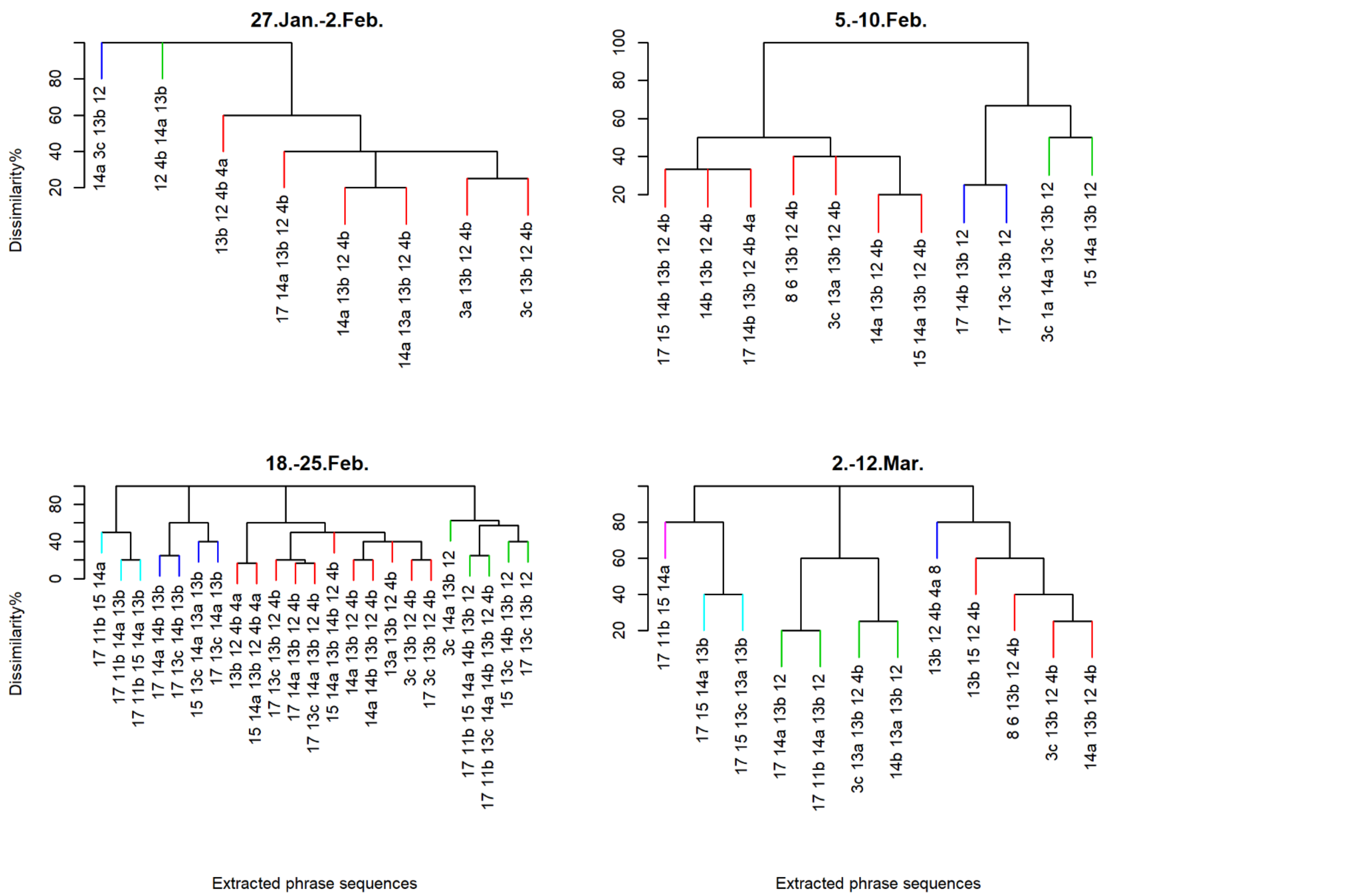

Fig G. Dissimilarity dendrograms for song sequences. The dissimilarity dendrograms show the song sequences which were delineated directly from the observed sound files for each period. Only sequences with a minimum of 4 different themes were included. The sequences connected with branches of the same color within each dendrogram represent a cluster of sequences which had a minimum of 40% similarity according to hierarchical clustering based on the Levenshtein Similarity Index (LSI)

According to the Levenshtein Similarity Index (LSI) 3-5 groups (with minimum of 40% similarity) of sequences were identified during each period with the greatest number of groups (5) during the last period, i.e. 2.-12. March (Table A). The mean Levenshtein Distance (LD) for each set median sequence (SM) was highest during that same period (Table A). From Fig G it is evident that the SM's were quite commonly observed within sequences though it varied what other more dynamic (shifting) themes were included. Perhaps, the SM for period 4 was the least representative for its period compared to the SM's of the previous three periods.

Table A. The representativity of each set median (SM) sequences. The table provides values which validate the representativity of each set median (SM) sequences for each period. The table provides the number of delineated sequences analysed, the number of different groups of theme sequences identified according to hierarchical clustering based on Levenshtein Distance values (LD)

Period	Set median sequence	No. different sequences	No. different sequence groups	Mean LD (±SD) of SM	Mean LD (±SD) of all sequences
1	14a-13b-12-4b	9	3	1.5 (1.9)	2.2 (1.4)
2	14a-13b-12-4b	11	3	2.2 (1.2)	2.5 (1.3)
3	17-14a-13b-12-4b	23	4	2.5 (1.3)	3.3 (1.4)
4	14a-13b-12-4b	12	5	2.7 (1.4)	3.2 (1.5)

Table B. Delineated song sequences of full song cycles. Delineated sequences of full song cycles captured within a 10 minute recording. The sequences are all from different sound files despite of some being detected during the same day. F = Full recorded phrase sequence within a 10-minute recording; T = Theme types in a full song which terminates when the start theme reoccurs; D = Delineated sequences according to the delineation protocol. Different delineated sequences are sepparated with a comma (,). Bold sequences are full songs according to the protocol and italic sequences are not full songs according to the protocol.

Date		The sequences captured within 10-minute recordings
31. Jan.	F	4b 13b 12 4b 12 4b 4b 4b
	T	4b-13b-12
	D	**13b-12-4b**
5. Feb.	F	13a 13a 13b 13b 13b 13b 12 12 4b 4b 4b 4b
	T	13a-13b-12-4b
	D	**13a-13b-12-4b**
10. Feb.	F	14b 14b 14b 13b 13b 13b 13b 12 12 4b 4b 4b 4b 4b 17 17 17 14b 14b 14b 13a 13a 13b 13b 13b 12
	T	14b-13b-12-4b-17
	D	**14b-13b-12-4b**, *17-14b-13a-13b-12*
18. Feb.	F	3c 3c 13a 13a 13b 13b 13b 13b 13b 13b 13b 13b 13b 13b
	T	3c-13a-13b
	D	**3c-13a-13b**
18. Feb.	F	3c 13b 13a 13b 13b 13b 12 12 4b 4b 4b 4b 17 17 17 13c 13c 13c 13b 13b 13b 13b 12 12
	T	13b-12-4b-17-13c
	D	*3c-13b 13a, 13b-12-4b, 17-13c-13b-12*
20. Feb.	F	17 14a 14a 14a 14b 14b 13b 12 12 4b 4b 4b 17 17 17 17 17 17 17 11b 11b 14a 14a 14a
	T	17-14a-14b-13b-12-4b
	D	**17-14a-14b-13b-12-4b**, *17-11b-14a*
20. Feb.	F	17 17 17 17 17 17 13c 13c 14b 14b 14b 14b 13b 13b 12 12 4b 4b 17 17 17 17
	T	17-13c-14b-13b-12-4b
	D	**17-13c-14b-13b-12-4b**
20. Feb.	F	13b 12 12 4b 4b 4b 17 17 17 17 17 17 17 17 17 13c 13c 13c 14a 14a 14a 14b 13b
	T	13b-12-4b-17-13c-14a-14b
	D	*13b-12-4b, 17-13c-14a-13b*
20. Feb.	F	13b 17 11b 11b 13c 13c 13c 13c 14a 14a 14a 14a 14a 14b 14b 14b 13b 13b 13b 12 12 12 4b 4b
	T	13b-17-11b-13c-14a-14b-13b
	D	**17-11b-13c-14a-14b-13b-12-4b**
24. Feb	F	13b 13b 12 12 12 12 4b 4b 4b 4b 4b 4b 17 17 17 17 14a 14a 14b 14b 14b 14b 13b
	T	13b-12-4b-17-14a-14b
	D	*13b-12-4b, 17-14a-14b-13b*
24. Feb	F	13b 13b 13b 12 12 4b 4b 4b 17 17 17 17 17 11b 11b 11b 14a 14a 14a 14a 14b 14a 13b 13b 13b
	T	13b-12-4b-17-11b-14a-14b
	D	*13b-12-4b, 17-11b-14a-14b-13b*
11. Mar.	F	13b 13b 14a 15 12 12 12 4b 4b 4b 4b 4b 17 17 15 15 15 14a 14a 14a 13a
	T	13b-14a-15-12-4b-17-15-14a-13a
	D	*13b-14a-15-12-4b, 17-15-14a-13*

Supplemental information on phrase sequence progression

Phrase sequence development within the subarctic songs

The smallest difference in phrase usage according to Dice's Similarity Index (DSI) was between period-1 and -2 (92% similarity), period-2 and -4 (97% similarity) and period-3 and -4 (93% similarity). The occurrence of the most common phrases, i.e. phrase-14a, 13b, 12 and 4b remained relatively stable between periods. The occurrence of phrase-3c gradually decreased while the occurrence of phrase-17 gradually increased from the first to the last period (Fig G). The occurrence of the less common phrases, such as phrase-6, -8, -4a, -13c and -11b, fluctuated more between periods (Fig 6). The most commonly observed themes were either composed of static or shifting phrases. The phrases 13b, 12 and 4b were static phrases which transitioned very consistently to a certain phrase in all periods while the shifting phrases 17, 13c, 14a and 3c transitioned to various phrases throughout the whole recording period (Table C). Other phrases were less common and contributed less to the transition variance.

Table C. Development in transitions of shifting phrases. Development in the transition of the relatively common, shifting phrases shown as a percentage of transitions obtained from Markov matrices. The prevalence of these phrases for each period are show as percentages of the total phrases observed during each period.

Shifting phrases	Period	% of total phrases	Transition behavior
Phrase-17	1	0.5%	**100% of the time to 14a**
	2	4.6%	**43% of the time to 13c**, 29% of the time to 15 and 29% of the time to 14b
	3	12.6%	**40% of the time to 11b,** 35% of the time to 13c and 20% of the time to 14a
	4	10.2%	**50% of the time to 15**, 25% of the time to 11b and 25% of the time to 14a
Phrase-13c	1	0.7%	No transition observed
	2	2.7%	**100% of the time to 13b**
	3	4.5%	**44% of the time to 14a**, 33% of the time to 13b and 22% of the time to 14b
	4	0.3%	**100% of the time to 13b**
Phrase-14a	1	21.7%	**70% of the time to 13b**, 15% of the time to 3c, 8% of the time to 8 and 6% of the time to 13a
	2	14.4%	**59% of the time to 13b**, 17.5 % of the time to 13a, 17.5% of the time to 3c and 6% of the time to 13c
	3	10.7%	**81% of the time to 13b**, 12% of the time to 14b and 8% of the time to 13a
	4	13.8%	**100% of the time to 13b**
Phrase-3c	1	9.9%	**69% of the time to 13b**, 15% of the time to 3c, 9% of the time to 8 and 6% of the time to 13a
	2	8.7%	**60% of the time to 8**, 20% of the time to 13b and 20% of the time to 11b
	3	3.6%	**70% of the time to 13b**, 20% of the time to 14b and 10% of the time to 8
	4	2.6%	**66% of the time to 8** and 33% of the time to 13b

Two transitional phrases were sometimes repeated once or twice by some singers before the transition completed. During these events the transitional phrases were categorized as regular phrases and assigned the phrase names '14b' and '13a', these are two versions of a typical transition between phrase-14a and -13b (Fig 4). These were the only transitional phrases observed to be repeated sequentially as if they were regular phrases.

The differences in signal quality between periods

Measurements were done on song units from the analysed sound files to investigate the different trend in the signal-to-noise ratio (SNR), the received signal level and the detection rate between periods.

A pairwise comparison using Wilcoxon rank sum test was conducted to estimate whether there was a statistical difference between periods for each measured value in Table 4. As a result, there was a significant difference between all periods in terms of the signal-to-noise ratio ($p<0.001$) though the least difference was found between period 3 and 4 ($P = 0.04$). The detection rate was statistically different between all periods ($p<0.001$) except periods 2 and 4. The received signal level was significantly similar between period 1 and 2 and between period 3 and 4, but significantly different between the first two and the two latter periods. That indicates that the whales were within a closer proximity to the recording station during the two latter periods (Table D).

These analysis show that the quality of the signals and the detection rate increased significantly in period 3 and continued to be relatively high in period 4. There was nonetheless a large variation in signal quality and detection rate during all periods, particularly during the 3rd period (18.-25. Feb). All analyzed sound files included full phrases with at least 10 dB SNR but some phrases had lower SNR. The lower SNR values were more common in the recordings from the first period (27. Jan-2. Feb) compared to the three following periods which suggests that some higher frequency phrases may have been lost (e.g. phrases 17 and 15) (Fig H).

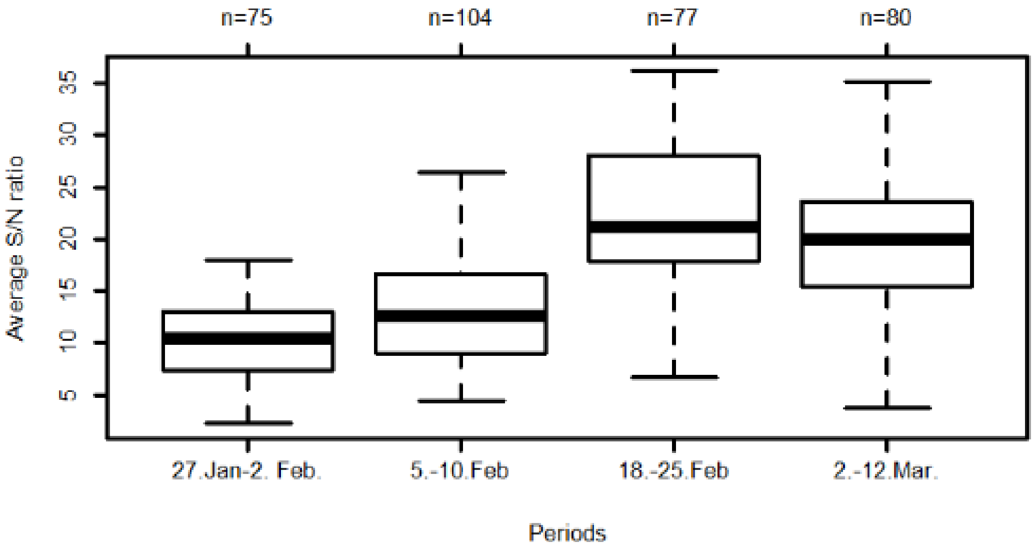

Fig H. The signal-to-noise ratio per period. The distribution of the signal to noise ratio of song units for each period obtained from the analyzed sound files.

Table D. The mean signal-to-noise level (SNR), the mean received signal level and the mean number of detections per analyzed sound file per period. In each analyzed sound files there were song units with a minimum of 10 dB above the background noise, however, some song units in the sound files could be lower and were included in the measurements, which explains the standard deviation (SD) for each period.

Period	Mean SNR (±SD)	Mean Received signal level (±SD)	Mean detections rate (±SD)	n
1	10.2 (4.1)	-51.7 (7.3)	43.4 (26.7)	75
2	13.0 (5.0)	-54.1 (5.3)	126.0 (55.9)	104
3	22.5 (7.2)	-37.9 (9.7)	230.1 (91.7)	77
4	19.8 (6.1)	-39.3 (10.7)	158.2 (42.2)	80
